# Poking Pluripotency: Nanoinjection Into Human iPSCs

**DOI:** 10.1002/adma.202521046

**Published:** 2026-03-14

**Authors:** Jann Harberts, Vuong Thi Thanh Xuan Ho, Yuqi Yang, Yuan Jiang, Roey Elnathan, Nicolas H. Voelcker

**Affiliations:** ^1^ Monash Institute of Pharmaceutical Sciences Monash University Parkville VIC Australia; ^2^ Melbourne Centre for Nanofabrication Victorian Node of the Australian National Fabrication Facility Clayton VIC Australia; ^3^ Materials Science and Engineering Monash University Clayton VIC Australia; ^4^ School of Medicine Faculty of Health Deakin University Waurn Ponds VIC Australia; ^5^ The Institute for Mental and Physical Health and Clinical Translation, School of Medicine Deakin University Waurn Ponds VIC Australia; ^6^ Institute For Frontier Materials Deakin University Waurn Ponds VIC Australia

**Keywords:** human induced pluripotent stem cells, mRNA, nanoinjection, nanoneedles, silicon nanotubes, transfection

## Abstract

Human induced pluripotent stem cells (hiPSCs) play an essential role in regenerative medicine and disease modeling, yet their utility critically depends on the efficient and safe delivery of exogenous genetic material. Conventional viral and electroporation‐based methods continue to present limitations, notably biosafety and regulatory barriers, suboptimal transfection precision, and substantial post‐transfection cytotoxicity. Here, for the first time, nanotube (NT)‐mediated nanoinjection is introduced as an effective non‐viral strategy for functional messenger RNA (mRNA) delivery into hiPSCs. To enable nanoinjection into hiPSCs, the work implements delayed extracellular matrix (ECM) application and advanced surface engineering, combined with a re‐engineered NT design featuring large cargo reservoirs to increase loading capacity and sharp rim geometries for improved cell interfacing. Effective nanoinjection into hiPSCs, while maintaining their pluripotent profile, is demonstrated using mCherry, GFP, and YPet mRNA (average transfection yields: ∼55% to ∼64%), including co‐transfection of mCherry and GFP mRNA (∼61%). Post‐nanoinjection hiPSC integrity is confirmed over several passages by excellent pluripotency marker expression (NANOG, OCT4, SOX2) and neuronal differentiation capacity. This proof‐of‐concept study demonstrates that nanoinjection is a powerful tool for delivering mRNA to hiPSCs with high expression yields and establishes nanoinjection as an enabling platform for precise cellular engineering and functional stem‐cell applications.

## Graphical abstract

Nanoinjection into hiPSCs: silicon nanotubes effectively transfect human induced pluripotent stem cells (hiPSCs) with mRNA, enabled by a delayed extracellular matrix application and enhanced surface functionalization. Nanoinjection is demonstrated with several reporter mRNA, including co‐transfection of mCherry and GFP. Importantly, the nanoinjected hiPSCs retain an excellent pluripotent profile for several passages after interfacing with the nanotubes.

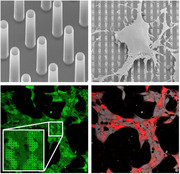

## 1 Introduction

Efficient cell engineering remains a major challenge in translational biomedical research [1, 2]. Among available cell types, human induced pluripotent stem cells (hiPSCs) provide an exceptional platform for disease modeling, drug discovery, and regenerative medicine owing to their dual capacity for indefinite self‐renewal and pluripotent differentiation [3, 4]. Importantly, their use in future personalized therapies and in vitro modeling of human development hinges on precise and reliable methods to perturb, reprogram, or edit gene expression—approaches required, for instance, to create specific human disease models [5]. Yet, hiPSCs are notoriously resistant to conventional transfection methods [6, 7, 8]. Electroporation and viral vectors, while commonly used, are associated with high initial toxicity, low post‐transfection viability, immunogenicity, and risks of genomic integration, limiting both the reproducibility and clinical applicability of engineered hiPSCs [9]. Lipid‐based systems, albeit gentler, often suffer from low efficiency and high variability [10]. Critically, the extreme sensitivity of hiPSCs to physical and chemical stressors further exacerbates these challenges, underscoring the need for efficient, non‐integrative, and minimally disruptive transfection technologies [11, 12]. Nanoinjection—the process of intracellular delivery using nanoneedles—is an emerging physical delivery route that efficiently transfects many cell types with spatial precision and retained cell integrity [13, 14].

Nanoneedle‐mediated nanoinjection uses high‐aspect‐ratio nanostructures, which can negotiate the cell membrane with minimal impact on cell viability and cellular function [15, 16, 17, 18, 19, 20]. Cargoes used in nanoinjection encompass a wide range of therapeutic agents, including small molecules, nucleic acids, proteins, and nanoparticles [21, 22, 23, 24, 25]. For cellular interfacing and cargo delivery, nanoneedle arrays have been fabricated in diverse configurations (e.g., nanostraws for nanoelectroporation) [26, 27, 28, 29, 30, 31, 32, 33, 34, 35] and from various materials, including silicon [36, 37, 38, 39, 40, 41, 42, 43], porous silicon [44], InP [45], InAs [46], GaAs [47], GaP [48], diamond [49], Si_3_N_4_ [50], ZnO [51], Al_2_O_3_ [31], PDMS [52], and polymers [53, 54]. But notably, silicon is a common choice due to well‐established nanofabrication methods that enable creating tunable nanoneedle geometries for optimized cell–nanoneedle interfacing [55, 56, 57, 58, 59]. Precise control over the cellular interface plays an important role in enabling cargo delivery, as high membrane curvatures correlate with the upregulation of endocytosis pathways [60, 61, 62, 63]. Beyond gene delivery, nanoneedle‐based systems have supported spatiotemporal lipidomics and RNA sampling [64, 65], influenced gene expression [66], regulated mechanotransduction machinery [67], and enhanced electrical interfacing of living cells [68], underscoring their versatility across future biomedical applications. However, extending nanoinjection to hiPSCs has remained elusive [13, 69].

The major challenges in enabling nanoinjection into hiPSCs stem from their stringent handling requirements and extreme sensitivity to external perturbations, often resulting in cell death or spontaneous differentiation [70]. This pronounced sensitivity further underpins their reputation as one of the most difficult‐to‐transfect cell types [6, 7, 8]. For establishing nanoinjection into hiPSCs, the implementation of correct handling steps is crucial for maintaining viability and pluripotency. A fundamental requirement for stable hiPSC growth is the presence of an extracellular matrix (ECM) on the substrate surface [71]. Without an ECM, hiPSCs fail to adhere, preventing growth on any cell culture ware, including nanoinjection substrates. However, established nanoinjection protocols do not use ECM coatings as they are not required for many other cell types. A further critical limitation in hiPSC culture is the need for hiPSCs to grow in compact colonies to maintain viability. To enable handling hiPSCs as single cells—as also required for nanoinjection—short‐term application of Rho‐associated protein kinase (ROCK) inhibitors is needed [72].

Here, we report a customized nanoinjection strategy enabling efficient transfection of hiPSCs with various types of reporter messenger RNA (mRNA), including co‐transfection, achieving average transfection yields of up to 64% with bright cytosolic expression, using a new type of nanotube (NT) array. In particular, the NTs featured large reservoirs for increased cargo loading per NT (reservoir volume ∼10× and surface area ∼4× larger than previous work) and sharply defined rims to improve cellular interfacing (<50 nm rim thickness). The nanoinjection chips were fabricated in a scalable top‐down approach using deep reactive ion etching (DRIE) of silicon wafers with an electron‐beam lithography (EBL)‐generated Cr hard mask. The sharp NT rims were achieved through a controlled undercut during dry etching, deliberately engineered to enhance membrane perturbation efficiency and hiPSC–NT interfacing. Homogeneous cargo loading was supported by surface functionalization using short‐chained poly‐D‐lysine (PDL, mol. wt.: 1–5 kDa), which proved essential for reliable mRNA expression by balancing loading and release. In addition to substrate optimization, we identified a key cell‐handling step that was critical for successful nanoinjection: A delayed application of the ECM prevented interference with the mRNA cargo, ensuring efficient uptake and high translational capacity of the delivered constructs. Furthermore, a ROCK inhibitor pretreatment enhanced hiPSC spreading and promoted intimate cell–NT interfacing, which was immediately established by gentle centrifugation. Following nanoinjection, hiPSCs reformed compact colonies with robust pluripotency marker expression (NANOG, OCT4, SOX2) and retained neuronal differentiation capacity (three passages post‐nanoinjection tested). Collectively, these advances establish a biocompatible and efficient nanoinjection platform for transfecting hiPSCs, providing a foundation for precise stem‐cell engineering.

## 2 Results and Discussion

### 2.1 Establishing a Nanoinjection Workflow Tailored for hiPSCs

**FIGURE 1:**
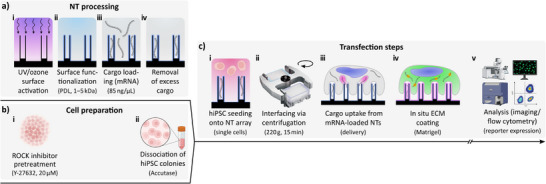
— Workflow of the NT‐mediated cargo delivery of mRNA to hiPSCs. Briefly, hiPSCs were interfaced with mRNA‐loaded NTs and analyzed the next day for the expression of a reporter protein (mCherry, GFP, YPet). a‐i) Surface activation with UV/ozone. a‐ii) Surface functionalization with poly‐D‐lysine (PDL, mol. wt.: 1–5 kDa). a‐iii) NT loading with mRNA (85 ng/µL). a‐iv) Removal of the cargo supernatant. b‐i) ROCK inhibitor preconditioning of the hiPSCs with 20 µM Y‐27632. b‐ii) Dissociation of the hiPSC colonies using Accutase. c‐i) hiPSC seeding onto the NT array (single cells). c‐ii) Centrifugation of cells onto NTs (220 g, 15 min). c‐iii) Cargo uptake from mRNA‐loaded NTs. c‐iv) In situ extracellular matrix (ECM) coating (Matrigel). c‐v) Analysis of expression using confocal microscopy imaging and flow cytometry (reporter proteins). Figure partly created with biorender.com.

The nanoinjection procedure was specifically tailored to the unique sensitivity and culture requirements of hiPSCs (Figure 1), enabling efficient mRNA transfection with robust reporter protein expression (mCherry, GFP, YPet), including co‐transfection. While nanoneedle‐mediated transfection has been demonstrated with various cell types, nanoinjection into hiPSCs has not been previously reported [69]. Key adaptations to existing nanoinjection protocols included: (i) a temporally delayed application of the ECM and (ii) a specific surface modification of the NTs, supported by a ROCK inhibitor preconditioning of the hiPSCs to promote cell handling and immediate centrifugation to facilitate rapid cell interfacing. In addition, a refined NT design was used, featuring a large cargo reservoir and sharp rims.

Specifically, the optimized nanoinjection workflow for transfecting hiPSCs with mRNA comprised three main phases: *NT processing* (Figure 1a), *cell preparation* (Figure 1b), and *transfection steps* (Figure 1c). The NT arrays were surface‐activated with a UV/ozone to increase wettability (Figure 1a‐i), and subsequently functionalized with short‐chained PDL (mol. wt.: 1–5 kDa) to generate a positively charged surface conducive to electrostatic mRNA adsorption (Figure 1a‐ii). mRNA was then loaded onto the NTs (Figure 1a‐iii), and excess cargo was removed just before cell seeding (Figure 1a‐iv). In parallel, hiPSCs were pretreated with a ROCK inhibitor for about 4 h to promote cell survival and spreading (Figure 1b‐i) before being dissociated into single cells using Accutase (Figure 1b‐ii).

For nanoinjection, single cells were seeded onto the mRNA‐loaded NTs (Figure 1c‐i) and immediately centrifuged to establish contact between the cell membrane and the NT tips (Figure 1c‐ii). Cells were incubated for 45 min to facilitate intracellular cargo delivery (Figure 1c‐iii), after which an ECM coating was applied to support overnight culture by adding pre‐diluted Matrigel to the cells (Figure 1c‐iv). hiPSCs were harvested on the next day for expression analysis using confocal microscopy and flow cytometry (Figure 1c‐v). Nanoinjection was tested with mRNA‐loaded NTs (NTs/mRNA), and control groups included flat Si with mRNA (flat/mRNA) as the topography control, and flat Si without mRNA (flat/blank) as the negative control. Importantly, NTs without mRNA showed no reporter expression analogue to flat/blank (Figure S1).

The in situ ECM coating (Matrigel), applied 1 h after cell seeding, played an important role in enabling the transfection, as the immediate presence of ECM proteins prevented hiPSC transfection (Figure S2, <1% when applied immediately). While ECMs are crucial for hiPSCs for adherence, growth, and expansion [71], we hypothesized that an ECM would impact cargo loading, delivery, and translation: the matrix proteins are likely to interfere with the mRNA, e.g., through mechanical entrapment or ECM‐associated mRNAse impurities. By first interfacing the hiPSCs with mRNA‐loaded NTs in the absence of any ECM, we temporally separated exposing the hiPSCs to mRNA from unwanted ECM interference so that the process of cargo uptake and later translation would be unimpaired. Such a delayed in situ ECM application is generally uncommon in standard hiPSC culture, but similar approaches have been described, for example, for in situ coating with laminin fragments or in situ Matrigel‐embedding of organoids [73, 74].

### 2.2 NT Fabrication, Design, and Functionalization

**FIGURE 2:**
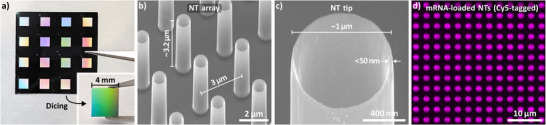
— NT array chip design, NT geometry, and cargo loading. a) Silicon wafer piece with 4×4 patterns, as used for DRIE processing. Inset: 4×4 mm^2^ wafer piece containing only NTs, as used for transfection experiments. b) Scanning electron microscopy (SEM) images of the NT arrays (array arrangement and NT dimensions). The array pitch was 3 µm, and the NT length was about 3.2 µm (large‐scale overview in Figure S6). c) Close up of the NT tip. The NT diameter was about 1 µm, and the rim thickness at the tip was less than 50 nm. d) Confocal microscopy images (top‐view) of NTs loaded with fluorescently‐labeled mRNA (Cy5‐tagged mRNA). The NT pattern was visible in the overview (dots), and steady Cy5 signal from the NTs illustrated homogeneous loading across the NT array (brightness quantification in Figure S9).

Briefly, the NTs were fabricated in a scalable top‐down approach using DRIE with a Cr hard mask (Figure S3). The rings that defined the NT structures were arranged on 4×4 mm^2^ areas using EBL at 4″ Si wafer scale with 8×8 patterns per wafer (Figure S4). Wafer pieces with 4×4 patterns were etched using DRIE (Figure 2a), and patterns were cut into 4×4 mm^2^ pieces to ensure exposing the hiPSCs only to NTs (inset). This was important since the cells were harvested from the NT arrays after transfection for further analysis. The geometry of the NT arrays used to mediate the transfection featured a NT length of ∼3.2 µm with an array pitch of 3 µm (Figure 2b). This pitch results in about 20 NTs per cell (assuming a cell diameter of 15 µm). The NT diameter was 1 µm with sharp rims of less than 50 nm at the NT tip (Figure 2c). After etching, the Cr hard mask initially remained (Figure S5), but Cr removal exposed the sharp rims, which resulted from a slight undercut right under the Cr layer. Notably, such sharp geometrical features used for nanoinjection are commonly only found for nanowire‐based nanoneedles, often with a conical shape [15, 17, 44, 49, 75, 76, 77, 78, 79, 80, 81, 82, 83]. Using scalable EBL and DRIE methods, the fabrication procedure allowed for creating large pattern areas with close to zero imperfections across the pattern area (Figure S6). Importantly, nanoneedles of similar geometry but without a cavity, thus lacking reservoirs and sharp rims, showed no transfection (Figure S7). This was in line with the literature reporting on the importance of sharp features for stimulating endocytosis pathways (200 nm curvature or less) [60, 61, 62, 63].

The cavity of the NTs accounted for about 75% of the NT length (Figure S8, cross‐section prepared by focused ion beam (FIB) milling). The cross‐section also showed a slight widening of the wall profile toward the base of the reservoir, which improved the mechanical stability of the NTs due to an increased material cross‐section [84]. The cavity added to the loading capabilities of the nanoneedles: a ’macroscopic’ reservoir and additional surface area. Compared with other work using shorter and smaller‐diameter NTs [42, 54, 85, 86, 87], the capacity of the reservoir was increased by about 7–10 fold from 0.1–0.14 µm^3^ (0.1–0.14 fL) to about 0.97 µm^3^ offering a larger amount of cargo available for delivery per NT (0.97 fL, assuming a frustum to compensate for the tapered walls; d_1_ = 600 nm, d_2_ = 800 nm, l = 2.5 µm). Accordingly, also the surface area of the cavity increased by a factor of about 4 compared to the smaller NTs [42, 54, 85, 86, 87], adding to the surface adsorption‐mediated loading capacity. Conventional nanowire‐based nanoneedles can load cargo merely by electrostatic binding to the outer nanoneedle surface (solid needles) or into small pores (porous Si needles) [17, 80]. These loading principles restrict the intrinsic loading capacity and cargo size: the loading capacity is predetermined by a limited surface area, thus providing only small amounts of cargo available for delivery, while the cargo size is restricted by the pore sizes of a few nm (only sufficient, e.g., for small‐sized siRNA but not mRNA). A ’macroscopic’ reservoir of hollow nanoneedles, however, offers larger loading capacity through increased volume and surface area, and also supports larger cargo sizes, including mRNA.

Effective cargo loading of the NTs was a crucial prerequisite for making cargo available for delivery. Loading of the NTs was supported by a surface functionalization step using PDL, which makes the surface electrostatically attractive to negatively charged mRNA. Confocal imaging of NTs loaded with cyanine 5 (Cy5)‐tagged mRNA showed a clean pattern of Cy5 signals from the NTs corresponding to their arrangement (Figure 2d), with a variation in the integrated brightness of only ±5.2%, underpinning homogeneous cargo loading (Figure S9). To rule out that the recorded intensity was caused by substrate reflections, the origin of the recorded signals was confirmed by intentionally bleaching the Cy5 dye using high‐power laser settings, which eliminated the Cy5 signal in exposed areas (Figure S10).

The molecular weight of the PDL used for surface functionalization (1–5 kDa) was also critical to achieve mRNA expression. Generally, surface functionalization of nanoneedles has been described before to support cargo loading—for example, for solid or porous nanoneedles—where the coatings included 3‐amino‐propyltriethoxysilane (APTES) [15, 76], polysaccharide‐polyplexes [79], polyethyleneimine (PEI) [82, 88], poly‐L‐lysine (PLL) [44, 83, 89], or PDL [17, 37, 53, 87]. While PDL and its enantiomer PLL indeed have been used before, we found that the standard mol. wt. commonly used to support cell adhesion (30–70 kDa) was ineffective for transfecting hiPSCs when used to functionalize the NTs (Figure S11, about 3.2% transfection). Notably, without PDL coating, transfection was also minimal, with just about 5% transfected cells, and showing only dim reporter signals (Figure S12). Only a low mol. wt. PDL coating enabled effective hiPSC transfection by balancing loading and release: generally, the stronger electrostatic binding of high mol. wt. PDL led to increased loading (brighter Cy5 signal, Figure S13a), but importantly, mRNA release from these substrates was reduced, indicating an excessive surface adsorption strength (Figure S13b). Successful release was further confirmed by imaging Cy5‐mRNA‐loaded NTs after 1 h exposure to cell culture medium, where for low mol. wt. PDL, the Cy5 signal vanished, while for high mol. wt. PDL, Cy5 signal remained with the NTs (Figure S13c).

### 2.3 Interfacing hiPSCs with mRNA‐Loaded NTs

**FIGURE 3:**
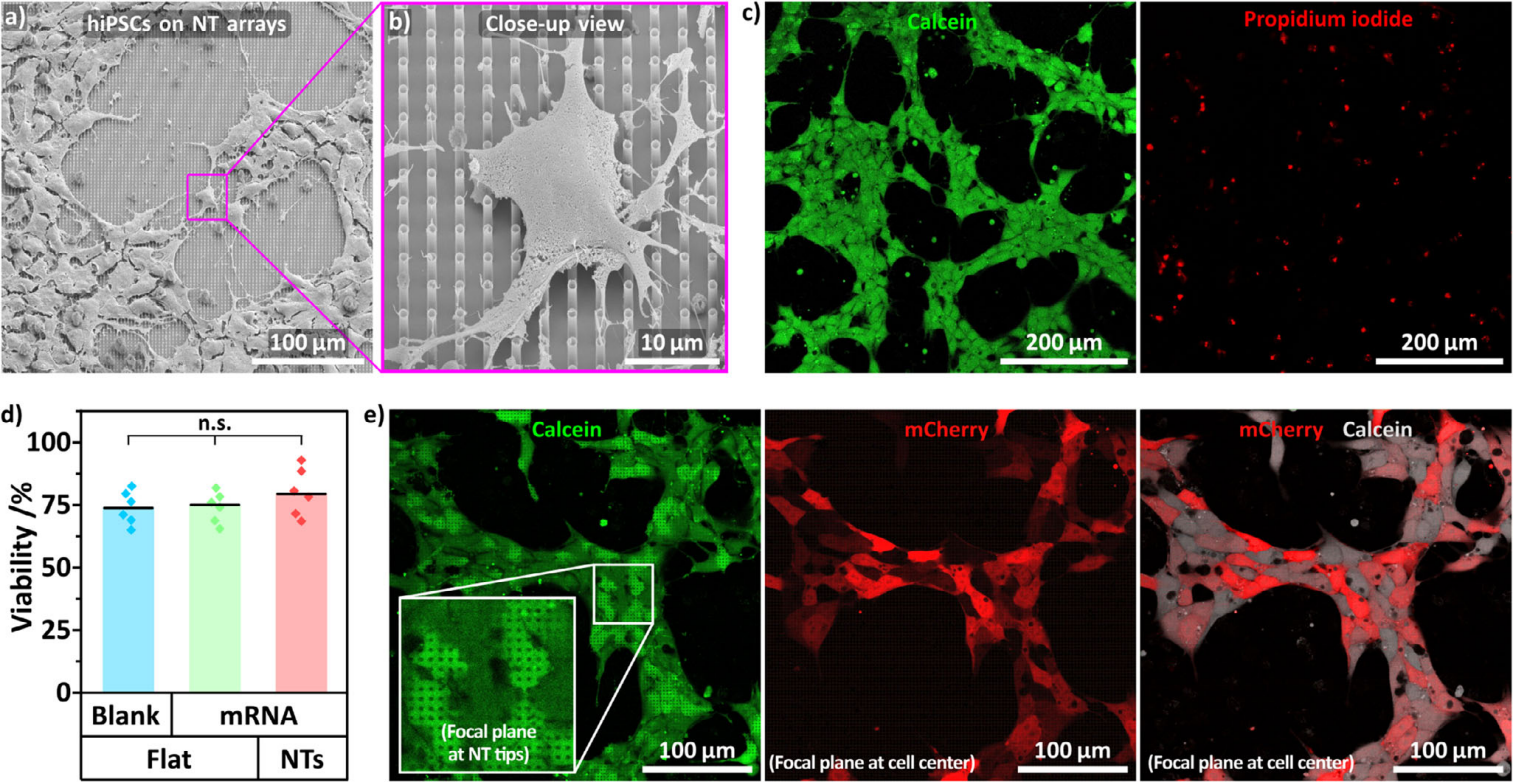
— hiPSCs interfaced with NT arrays imaged with SEM and confocal microscopy. a) Overview SEM image of hiPSCs cultured on the NT arrays (additional examples in Figure S18). b) Close‐up view of an individual cell (magnified from panel a). c) Viability staining of hiPSCs cultured overnight on NTs (viable cells stained with Calcein (Ca), green; dead cells stained with propidium iodide (PI), red). Hoechst 33342 nuclear counterstain and flat controls shown in Figure S19a. d) Cell viability of hiPSCs cultured on flat/blank, flat/mRNA, and NTs/mRNA derived from the PI channel (Ca‐derived cell viability in Figure S19b). e) Confocal microscopy images of hiPSCs on mCherry‐mRNA‐loaded NTs before harvesting and while expressing mCherry, red (cells stained with Ca, green/gray). Inset: The NT array was visible by dark spot pattern in the cytosol, indicating indentations of the cell membrane (focal plane at the NT tips). mCherry was imaged with the focal plane at the cell center (further away from the NTs). Additional examples in Figure S20. ANOVA with post hoc Tukey's test, n.s.: non‐significant, *n* = 3.

For nanoinjection, hiPSCs were dissociated and seeded onto the NT arrays supported by centrifugation in order to prompt interfacing the cells with the cargo‐loaded NTs. Immediate centrifugation after cell seeding within 1 min was important to maximize transfection by minimizing premature cargo release. Notably, a 5‐min delay of the centrifugation step led to reduced transfection, similar to seeding without centrifugation (∼34% transfection, Figure S14). Nanoinjection was conducted with 6–7 day‐old colonies, which grew in a densely packed formation with well‐defined edges and without spontaneously differentiated cells (Figure S15). A short ROCK inhibitor pretreatment was used to precondition the cells before being exposed to the NT arrays to ease cell handling (20 µM, 4 h; Figure S16, visualization of rougher colony edges). The application of ROCK inhibitors, including pretreatment [90], is common in stem cell culture and required to enable single‐cell handling of hiPSCs, as this sensitive cell type has poor cell survival outside its regular colony growth [72]. While a concentration of 10 µM is conventionally used to maintain cell survival, higher concentrations have been tested to further support stem cell survival and proliferation [91]. Here, we chose an above‐average ROCK inhibitor concentration, since the ROCK‐induced actomyosin suppression also causes increased cell spreading (up to three times higher than untreated hiPSCs) [92], which we hypothesized would enhance interaction with the NTs. The ROCK inhibitor treatment was performed ahead of the transfection experiment to ensure the cells were already affected by the ROCK inhibitor when seeded onto the NT arrays. While transfection was still possible without pretreatment (Figure S17, about 45% transfection), we found cell handling and transfection more reliable with pretreatment. ROCK inhibitor‐treated hiPSCs cultured overnight on NT arrays spread uniformly across the chips despite the sharp substrate topography (Figure 3a; close‐up view in Figure 3b; additional examples in Figure S18).

Cell viability of hiPSCs cultured overnight on NTs was assessed with Calcein (Ca) and propidium iodide (PI) staining (Figure 3c, Hoechst 33342 nuclear counterstain and flat controls in Figure S19a). hiPSC viability on NTs was about 75% similar to flat controls (Figure 3d, PI‐derived cell viability; Ca‐derived in Figure S19b). Notably, minimal impact of the NTs on cell viability is in line with several studies testing the biocompatibility of nanoneedle arrays [39, 40, 46, 85, 93].

High‐resolution confocal microscopy imaging of hiPSCs interfaced with NTs showed membrane indentations corresponding to the NT positions, visible by dark puncta in the Ca signal, where the NTs displaced the cytosol (Figure 3e, inset in Ca channel). Imaging of hiPSCs on NTs also showed the mCherry expression in the cells with homogeneous signal distribution in the cytosol (mCherry channel in Figure 3e, additional examples in Figure S20). Notably, the hiPSCs only wrapped around the upper part of the NTs without making contact with the substrate bottom (Figure S21, SEM images of FIB‐milled cell–NT cross‐sections) [94].

### 2.4 Nanoinjection of Various mNRA Types, Including Co‐Transfection

**FIGURE 4:**
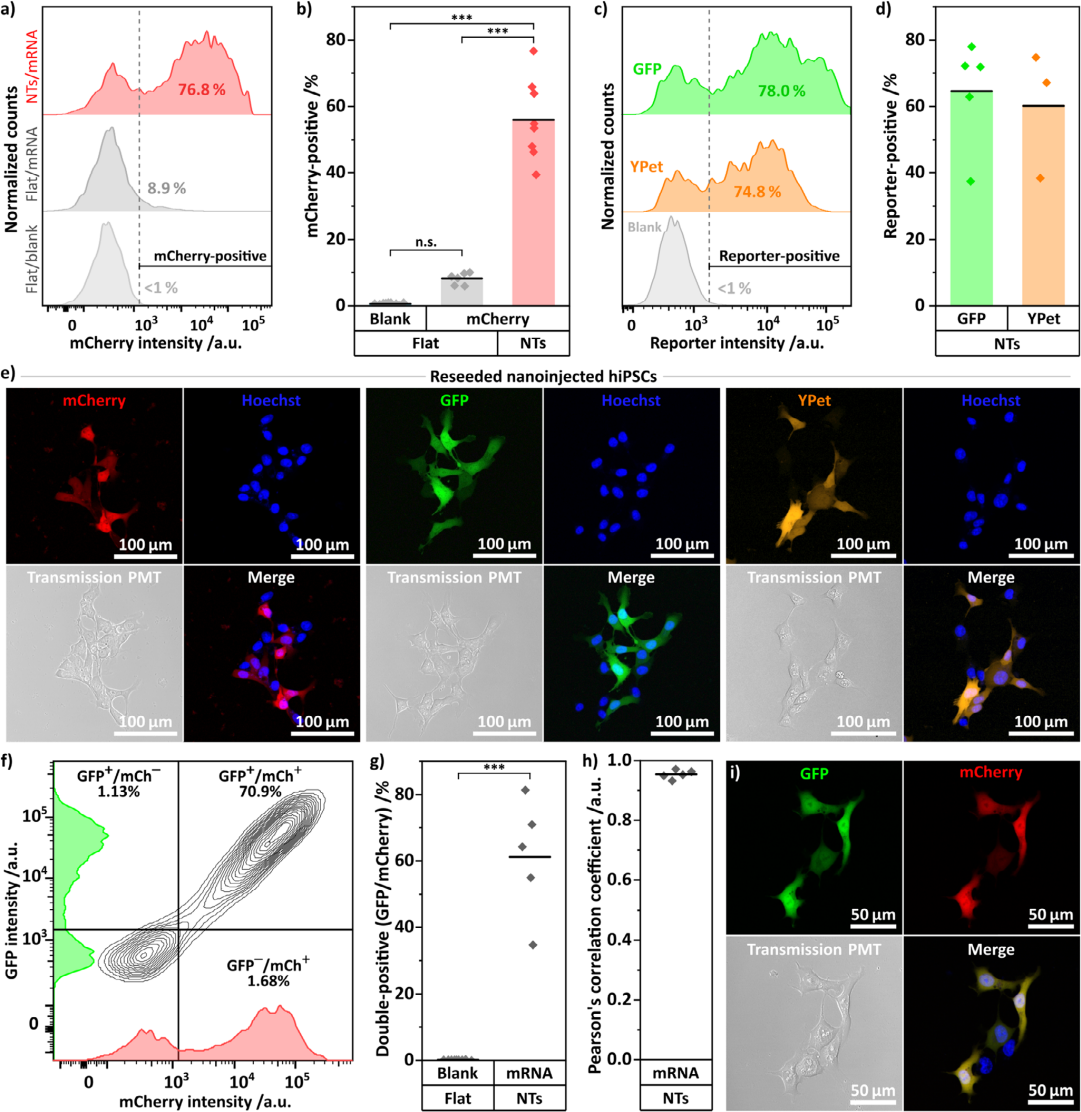
— Nanoinjection of various mRNA constructs into hiPSCs, including co‐transfection. Quantification of transfection yield via flow cytometry analysis and cell visualization via confocal microscopy. a) Exemplary flow cytometry plots (histograms of mCherry intensity) of hiPSCs harvested from blank, i.e., non‐loaded, flat silicon chips (flat/blank, negative control, light gray), cargo‐loaded flat silicon chips (flat/mRNA, topography control, gray), and cargo‐loaded NT arrays (NTs/mRNA, red), cargo: mCherry‐expressing mRNA. b) Proportion of mCherry‐positive cells harvested from flat/blank, flat/mNRA, and NTs/mRNA, cargo: mCherry‐expressing mRNA. Quantification via flow cytometry; mCherry intensity gated to <1% of cells harvested from flat/blank silicon (*n* = 8, entire gating stream including back‐gating in Figure S22a). c) Exemplary intensity plots of hiPSCs nanoinjected with GFP and YPet mRNA. d) Transfection yields using GFP and YPet mRNA, *n* = 3–5. e) Exemplary confocal microscopy images of hiPSCs harvested from NT arrays after NT‐transfection with mCherry/GFP/YPet‐expressing mRNA, and replated onto Matrigel‐coated imaging dishes (10 µM Y‐27632 ROCK inhibitor, ∼4 h after seeding). The reporter‐positive hiPSCs showed homogeneous distribution of reporter fluorescence inside the cytosol with varying intensities between different cells (nuclear counterstain: Hoechst 33342). f) Exemplary mCherry/GFP intensity plot of co‐nanoinjected hiPSCs. g) Quantification of double‐positive hiPSCs. *n* = 5. h) Quantification of Pearson's correlation coefficient of double‐positive hiPSCs. i) Exemplary confocal images of reseeded co‐nanoinjected hiPSCs, including merge of mCherry and GFP channel (additional examples in Figure S26). All intensity histograms used for quantification are compiled in Figure S27. ANOVA with post hoc Tukey's test, n.s.: non‐significant, *** *p* < 0.001, *n* = 3–8.

Following nanoinjection, hiPSCs were harvested for quantitative transfection analysis via flow cytometry. First, we tested the delivery of mCherry mRNA: nanoinjected hiPSCs showed a distinct population of *bright* mCherry‐expressing cells (Figure 4a), confirming high expression levels and indicating high mRNA doses delivered, as dose and expression correlate linearly [95]. The gating threshold for reporter fluorescence was set to <1% positive events in the blank control (flat/blank). Back‐gating of the mCherry‐positive cells confirmed a homogeneous distribution in the main population plot, indicating non‐selective transfection of the hiPSCs (Figure S22).

Quantitative analysis revealed that nanoinjection yielded ∼56% mCherry‐positive cells on average, which was in contrast to a mean of 8.2% for flat controls (Figure 4b). Importantly, the small fraction of mCherry‐positive cells on flat controls originated only from dim, low‐intensity events, as evident from the fluorescence histograms and geometric mean values of mCherry brightness (Figure S23a, flats about 7 fold lower than NTs). Overall, the NT‐based nanoinjection achieved an about 54‐fold enhancement in transfection performance compared with flat controls, as reflected by the expression index (Figure S23b; geometrical mean × proportion positive). Next, we tested GFP and YPet mRNA to demonstrate that nanoinjection is compatible with different mRNA types (Figure 4c), including mCherry and GFP mRNA using a different capping and nucleotide modification technology (Figure S24a; mRNA details compiled in the methods). GFP and YPet showed similar transfection yields (Figure 4d, ∼64% and ∼60%, respectively), and so did the other mCherry and GFP variants (Figure S24b, ∼55% and ∼57%, respectively). Confocal microscopy of reseeded nanoinjected cells displayed uniform cytosolic mCherry/GFP/YPet signal with varying intensity among individual cells (Figure 4e; cells were briefly incubated for about 4 h before confocal microscopy imaging; nuclear counterstain: Hoechst 33342, additional close‐ups in Figure S25). In addition to single‐vector transfection, the co‐delivery of two mRNAs was tested: nanoinjection into hiPSCs was capable of transfecting with mCherry and GFP mRNA simultaneously (Figure 4f). Quantification of double‐positive cells showed similar transfection yields compared to single transfection (Figure 4g, ∼61%). Importantly, transfection of mCherry and GFP was highly balanced, indicated by a Pearson's correlation coefficient of about 0.95 (Figure 4h). Co‐transfected cells also showed homogeneous expression in the cytosol (Figure 4i, additional examples in Figure S26).

### 2.5 Post‐Nanoinjection Integrity of the hiPSCs

**FIGURE 5:**
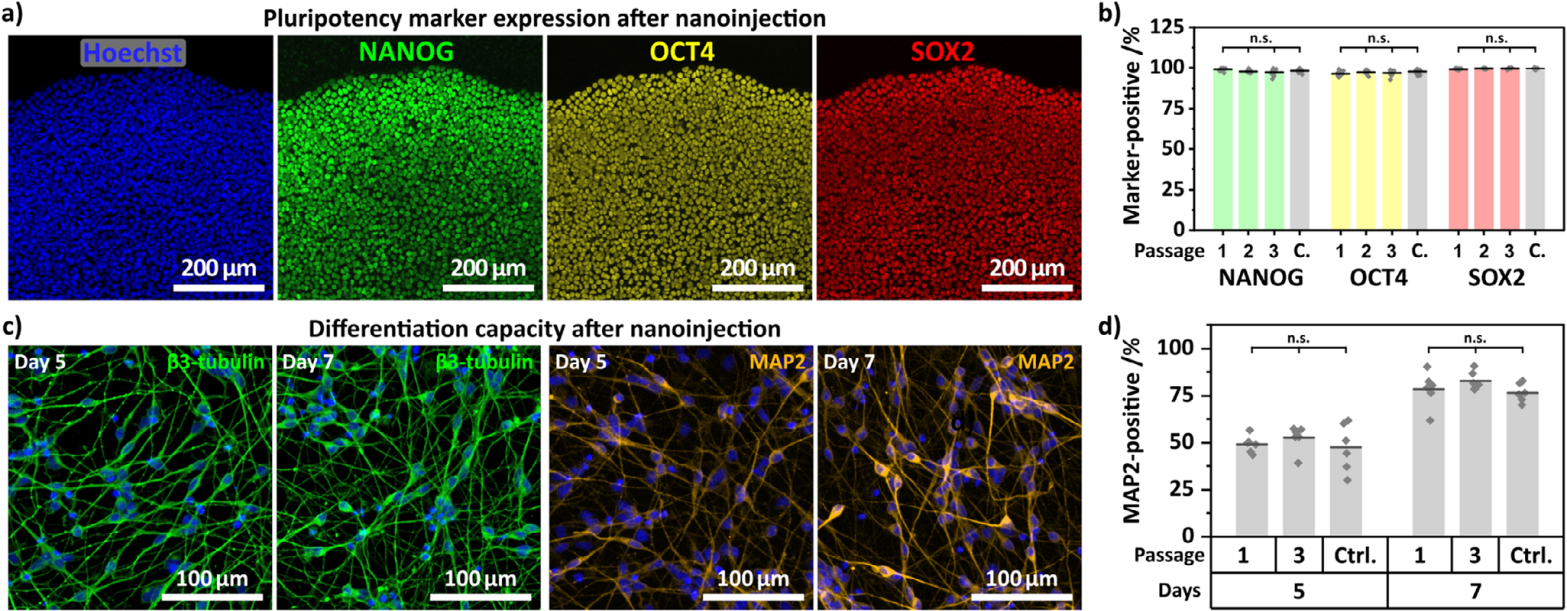
— Post‐nanoinjection cell quality: pluripotency profile and differentiation capacity. a) Exemplary confocal microscopy images of immunostained hiPSCs cultured for 3 passages (∼3 weeks) after nanoinjection (nuclear counterstain: Hoechst 33342, blue; anti‐NANOG, green; anti‐OCT4, yellow; anti‐SOX2, red). Daily brightfield imaging of the colony growth in Figure S28, including flat controls and regularly passaged hiPSCs. Passage 1 and 2, including regularly passaged control in Figure S30. b) Quantification of NANOG‐, OCT4‐, and SOX2‐positive cells for passage 1–3, including regularly passaged hiPSCs (C.). *n* = 4. c) Exemplary confocal microscopy images of neurons generated from nanoinjected hiPSCs (passage 1) stained for β3‐tubulin and MAP2 imaged at day 5 and 7 of the neuronal differentiation protocol (passage 3 and controls in Figure S32). d) Quantification of MAP2‐positive neurons generated from passage 1 and 3 nanoinjected hiPSCs compared to regularly passaged controls. *n* = 3. ANOVA with post hoc Tukey's test, n.s.: non‐significant. *n* = 3–4.

hiPSCs harvested from NTs readily reformed compact colonies, with well‐defined edges and growth behavior comparable to cells harvested from flat controls and regularly passaged hiPSCs (Figure S28; 5‐days timeline). Immunostaining of these cells further confirmed sustained expression of pluripotency markers NANOG and OCT4 (Figure S29a), with similar proportions of marker‐positive cells compared to flat controls and regular passaging (Figure S29b, close to 100% marker‐positive cells). We maintained nanoinjected hiPSCs for three passages (∼3 weeks) and checked pluripotency marker expression of NANOG, OCT4, and SOX2 for every passage (passage 3 in Figure 5a; passage 1 and 2 as well as control in Figure S30). Quantification showed stable marker expression for the tested passages (Figure 5b, close to 100% marker‐positive cells).

Additionally, we tested the differentiation capacity of the nanoinjected hiPSCs (passage 1 and 3) toward a neuronal cell fate, and following a neuronal differentiation protocol, the cells showed neuronal morphology (Figure S31). Immunocytochemistry staining for the neuronal markers β3‐tubulin and MAP2 confirmed successful neurogenesis of the nanoinjection hiPSCs (Figure 5c, passage 1; passage 3 and controls in Figure S32). Marker quantification showed similar proportions of MAP2‐positive cells for passage 1 and 3 compared to the controls (Figure 5d, ∼80% at day 7) and the literature [96]. Unimpaired post‐transfection integrity (colony formation, expression of pluripotency markers for several passages, and neuronal differentiation capacity) of nanoinjected hiPSCs confirmed the applicability of nanoinjection into hiPSCs for use in future cellular applications.

## 3 Conclusions

We demonstrate the first effective nanoinjection of mRNA into hiPSCs, including co‐transfection, with bright cytosolic fluorescence, indicating high expression levels relevant for downstream applications. Average transfection yields were between ∼55% and ∼64% for different reporter mRNAs (mCherry/GFP/YPet), and ∼61% for co‐nanoinjected mCherry/GFP (double‐positive hiPSCs). Successful nanoinjection into hiPSCs was enabled by integrating key processing and handling steps into existing nanoinjection protocols. These important changes included: (i) a delayed in situ application of the ECM to prevent interference with cargo delivery (1 h after seeding) and (ii) a surface functionalization of the NTs with a low molecular weight PDL (1–5 kDa) to balance loading and release. Effective nanoinjection was supported by a ROCK inhibitor pretreatment of the hiPSCs to enhance cell spreading and NT interfacing (4 h, 20 µM) and an immediate centrifugation step to promote rapid hiPSC‐NT interfacing. hiPSCs exposed to NTs retained ∼75% viability, similar to flat controls, confirming biocompatibility of the nanoinjection substrates. Post‐transfection, nanoinjected cells reformed compact colonies, retained expression of the pluripotency markers NANOG, OCT4, and SOX2, and maintained the capacity for neuronal differentiation, underscoring the platform's suitability for continued culture and downstream applications. This proof‐of‐concept study establishes nanoinjection as a novel strategy to precisely manipulate hiPSCs with mRNA‐encoded transcription factors, and the ability to achieve high‐efficiency transfection without compromising cell integrity opens up new avenues for stem‐cell engineering.

## 4 Experimental Section

### 4.1 NT Fabrication

Briefly, the NTs were fabricated with a top‐down approach from Si wafers using a Cr hard mask (Figure S3). Specifically, Si wafers (Siltronix, Boron p‐doped, 3–6 Ω cm, <100>) were dehydrated (3 min, 180°C) and spin‐coated with about 75 nm thick poly(methyl methacrylate) (PMMA, A2), including post‐bake (3 min, 180°C). Ring patterns (outer ring diameter: 1 µm, wall thickness: 160 nm, array pitch: 3 µm, pattern area: 4×4 mm^2^; 8×8 pattern per 4″ wafer, Figure S4) were defined using EBL (Vistec EBPG‐5000+, Raith; 100 V, 40 nA beam current, 20 nm beam step size, 1000 µC/cm^2^). Exposed ring patterns were developed for 1 min in 3:1 methyl isobutyl ketone (MIBK):isopropyl alcohol (IPA), stopped for 15 s in IPA, rinsed with deionized (DI) water, and dried (N_2_ blow). The Cr hard mask (22 nm thickness) was deposited using e‐beam evaporation (AMOD system, Angstrom Engineering), including a 15 s Ar plasma before deposition to enhance adhesion. Lift off was done using Acetone with brief sonication (10 s), followed by IPA and DI rinse, and N_2_ blow drying. NTs were created using DRIE (PlasmaPro 100 Estrelas, Oxford Instruments) in a pseudo‐Bosch process (SF_6_: 20 sccm, C_4_F_8_: 19 sccm, pressure: 10 mTorr, table RF power: 40 W, ICP RF power: 1200 W, carrier temperature: 10°C, etch rate: about 730 nm/min). Etched NTs were post‐processed with an O_2_ plasma cleaning (O_2_: 50 sccm, pressure: 10 mTorr, table RF power: 15 W, ICP RF power: 1000 W, 2 min). Lastly, the Cr mask was removed using Cr etchant (wet etching, 5 min at room temperature, RT).

### 4.2 hiPSC Culture

Briefly, the hiPSCs were maintained on Matrigel‐coated well plates using mTeSR+ medium. Specifically, the hiPSC line (BIONi010‐C‐13, batch/lot No.: M001) was purchased from the European Bank for Induced Pluripotent Stem Cells (EBiSC.org) [97]. Experiments were conducted with 6–7 day‐old colonies. Cells were passaged weekly using ReLeSR (#100‐0483, StemCell Technologies, 5–6 min incubation at RT) at split ratios of 1:50–1:100. The mTeSR+ stem cell culture medium (#100‐0276, Stem Cell Technologies) was changed every 2–3 days. Well plates were coated overnight at RT with Matrigel (Corning, #354263, approx. 19 mg/mL, prediluted at 1:5 in Knockout DMEM, #10829018, ThermoFisher Scientific, final dilution of 1:150) using 1.5 mL per well of a 6‐well plate. Cells were kept at 37°C and 5% CO_2_ in the incubator.

### 4.3 Cargo Loading of Si Chips (Flat and NTs)

Briefly, the NT arrays were functionalized to support cargo loading, and cargo was loaded by incubation with mRNA solution. Specifically, silicon chips (NT arrays and flat controls) were treated for 40 min with UV/ozone (Samco UV Ozone Cleaner) to increase hydrophilicity. Using 48‐well plates (easier sample handling), samples were submerged in 80% EtOH for sterilization (10 min), and rinsed 3× with UltraPure DNase/RNase‐Free Distilled Water (UP DI, #10977015, Invitrogen, ThermoFisher Scientific). Substrate surfaces were functionalized (30 min in vacuum) with poly‐D‐lysine (PDL, #P0296, Sigma Aldrich, mol. wt. 1,000–5,000 Da) using 6 µL (0.1 mg/mL) per chip (4×4 mm^2^), held on the chip by surface tension. PDL solution was removed, chips fully dried in vacuum (10 min), rinsed 3× with UP DI (to remove excessive PDL), moved to 96‐well plates (to enable multi‐channel pipetting), and fully air‐dried. Notably, without drying of the PDL before rinsing, transfection was less consistent (Figure S33). mRNA was diluted to about 85 ng/µL using UP DI, pipetted onto the chips (4.5 µL/chip, held on the chip by surface tension), kept on ice (to maintain mRNA integrity and minimize evaporation), and placed on an orbital shaker (45 rpm), while samples were incubated for 1 h. Used mRNA was: mCherry‐encoding mRNA (#L‐7203, TriLink, CleanCap, 5moU, 1 µg/µL), eGFP‐encoding mRNA (#L‐7601, TriLink, CleanCap, 5mU, 1 µg/µL), YPet‐encoding mRNA (#130‐120‐971, Miltenyi Biotech, capped, pseudouridine and 5‐methyl‐cytidine, lyophilized: 20 µg, reconstituted: 500 ng/µL), mCherry‐encoding mRNA (Messenger Bio, Cap1, N1‐methylpseudouridine, 1 µg/µL), and eGFP‐encoding mRNA (#130‐101‐114, Miltenyi Biotech, capped, pseudouridine and 5‐methyl‐cytidine, lyophilized: 20 µg, reconstituted: 500 ng/µL). Blank controls were kept mRNA‐free. For co‐transfection, a mixture of mCherry and GFP mRNA was used for loading. Note, for visualizing the cargo loading via confocal microscopy (Leica Stellaris 5), a Cy5‐tagged mRNA variant was used, and brightness was analyzed with CellProfiler. Just before cell seeding, the mRNA solution was removed, leaving a thin film behind to keep the chips moisturized until adding the cell suspension.

### 4.4 Release Test

Chips were loaded with mRNA as described above (Cy5‐tagged where applicable). Subsequently, chips were briefly submerged in 1 mL PBS (#10010‐023, Gibco, ThermoFisher Scientific) to remove excess cargo, tipped onto a tissue to remove moisture from the bottom, and placed into a well of a 24‐well plate. Chips were immediately covered with 4.5 µL PBS and incubated for 0, 1, 5, and 60 min (for 0 min, the PBS was removed immediately). mRNA concentrations in the supernatants were determined using a NanoDrop 2000c spectrophotometer (ThermoFisher Scientific).

### 4.5 Transfection Procedure

Briefly, hiPSCs were seeded onto cargo‐loaded chips and cultured overnight. Specifically, hiPSCs were pretreated with 20 µM Y‐27632 (#130‐103‐922, StemMACS, Miltenyi Biotec) for 4 h. Colonies were dissociated into single cells using Accutase (#07920, StemCell Technologies) for 15 min at 37°C. Cells were counted, centrifuged (300 g, 5 min), and resuspended in Opti‐MEM (#31985062, Gibco, ThermoFisher Scientific) supplemented with 20 µM Y‐27632 at 350,000 cells/mL. Cells were seeded onto the mRNA‐loaded and blank Si chips (150 µL/well of 96‐well plate, i.e., ∼165,000 cells/cm^2^) and well plates with the Si chips were immediately centrifuged (220 g, 6/3 acceleration/deceleration, 35°C, 15 min) to establish the cell–NT interface. For seeding multiple wells simultaneously, an electronic multichannel pipette was used (Picus, 12‐ch, 50–1200 µL, Sartorius, lowest dispensing speed). Centrifuged chips were placed for 45 min in the incubator. Prediluted Matrigel (1:5) was added to the cells (5 µL/well, final concentration: 130 µg/mL) and samples incubated for an additional 1 h in the incubator. Matrigel‐containing Opti‐MEM was replaced with mTeSR+ medium supplemented with 10 µM Y‐27632, and samples were kept overnight in the incubator.

### 4.6 Fluorescence Imaging of Cells on NT/Flat Chips (Viability and mCherry Expression)

hiPSCs on NTs were prepared analogue to the transfection experiments on flat/blank, flat/mRNA, and NTs/mRNA. For assessing the cell viability on the chips, cells were stained with Calcein‐AM (Ca, viable cells, #C1359, Sigma Aldrich, 0.5 µM), propidium iodide (PI, dead cells, #P4864, Sigma Aldrich, 0.5 µg/ml), and Hoechst 33342 (#H1399, ThermoFisher Scientific, 20 µg/mL) in mTeSR+ for 5 min at 37°C. Cells were rinsed with fresh mTeSR+ and immediately imaged using confocal microscopy. Viability was derived from both Ca and PI (Ca‐positive/Hoechst, (Hoechst – PI‐positive)/Hoechst). For visualizing the mCherry expression of cells interfaced with NTs, the cells were stained with Ca, and imaged with confocal microscopy.

### 4.7 Scanning Electron Microscopy Imaging of hiPSCs on NTs

hiPSCs on NTs/mRNA chips were prepared analogue to the transfection experiments. Instead of harvesting, the cells were fixed 2 h at RT and overnight at 4°C with a mix of 2% paraformaldehyde (ProSciTech, #C006, 8%) and 1.9% glutaraldehyde (#C16537‐16, PriSciTech, 2.5% aqueous solution, EM grade), stocks mixed 1:4. Samples were rinsed 3× with UP DI, followed by a stepwise ethanol exchange (25%, 50%, 75%, 90%, 2×100%, each 10 min). Ethanol was exchanged with 50% hexamethyldisilazane (HMDS, Sigma Aldrich, #33350‐U) in ethanol and 2×100% HMDS, each for 10 min. HMDS was removed, and chips were air‐dried. For imaging and FIB‐milling (FEI Helios Nanolab 600 Dual FIB‐SEM), samples were sputtercoated with 10 nm Au (Emitech K550X sputtercoater).

### 4.8 Flow Cytometry Analysis of Harvested Cells

Briefly, cells were harvested from the samples and processed for flow cytometry analysis. Specifically, two extra wells per chip were prepared with 100 µL Accutase. Chips were submerged in the first well for rinsing, and placed in the second well for cell detachment (15 min, 37°C). Accutase was diluted with 50 µL mTeSR+ medium, and the cell suspension was transferred to v‐bottom 96‐well plates for centrifugation (450 g, 5 min, 4°C). Medium was discarded and cells were resuspended in fluorescence‐activated cell sorter (FACS) buffer (Dulbecco's phosphate‐buffered saline (DPBS, –Ca^2+^, –Mg^2+^), 2 mM ethylenediaminetetraacetic acid (EDTA), 0.1% NaN_3_) supplemented with LIVE/DEAD Fixable Aqua Dead Cell Stain (LD, 1:1000, #L34966, Invitrogen) as dead stain (10 min, RT, see Figure S22b for stain controls). Cells were washed once with FACS buffer and kept in FACS buffer with 2% fetal bovine serum (FBS) on ice until flow cytometry analysis (BD LSRFortessa X‐20 cell analyzer). Cells were gated by population (FSC‐A × SSC‐A), singlets (FSC‐A × FSC‐H), live (FSC‐A × LD), and reporter expression (intensity histograms, mCherry/GFP/YPet) (Figure S22a). mCherry, GFP, and YPet were gated to <1% reporter‐positive cells using the flat/blank controls. GFP/YPet and LD were compensated accordingly (Figure S22c).

### 4.9 Imaging of Reseeded Cells

Cells were harvested from the chips analogous to the flow cytometry analysis, but seeded into Matrigel‐coated culture ware. For mCherry/GFP/YPet visualization, cells were seeded into Matrigel‐coated (1 h@37°C) imaging Petri dishes (FluoroDish, #FD35‐100, World Precision Instruments) or imaging chambers (µ‐slide 8 well, high, ibiTreat, Ibidi, #80806) using mTeSR+ supplemented with 10 µM Y‐27632. Cells were incubated for ∼4 h to allow for cell adhesion before imaging. Nuclei were stained with Hoechst 33342 (20 µg/mL, 5 min, 37°C), and live cells were imaged using confocal microscopy. For long‐term culture, harvested cells were seeded into 24‐well plates using mTeSR+ supplemented with 10 µM Y‐27632. Y‐27632 was withdrawn the next day, and hiPSCs were cultured for three passages (∼3 weeks). Neuronal differentiation was initiated from these cells as described in the respective section. Cells were fixed at the respective time points with 4% paraformaldehyde (ProSciTech, #C006, 8% diluted 1:1 in mTeSR+) for 15 min at RT. Cells were washed 3× with DPBS, and the well plates were stored at 4°C until further use. Samples were permeabilized and blocked (3% bovine serum albumin, BSA, ThermoFisher Scientific, #A8412; 0.2% Triton X‐100, Sigma Aldrich, #T8787; 0.1% Tween‐20, Sigma Aldrich, #P2287; in DPBS) for 45 min at RT. Primary pluripotency antibodies (Abcam, mouse anti‐NANOG, #ab173368, 1:200; rabbit anti‐OCT4, #ab181557, 1:250; goat anti‐SOX2, #ab239218, 1:250) and neuronal antibodies (mouse anti‐β‐tubulin 3, Sigma Aldrich, #T8578, 1:500; mouse anti‐MAP2, ThermoFisher Scientific, #13‐1500, 1:500) in DPBS with 0.1% BSA were incubated overnight at 4°C. Samples were washed 2× with DPBS and secondary antibodies (ThermoFisher Scientific, goat anti‐mouse Alexa Fluor 488, #A11029; goat anti‐rabbit Alexa Fluor Plus 555, #A32732; donkey anti‐mouse Alexa Fluor Plus 488, #A32766; donkey anti‐rabbit Alexa Fluor Plus 555, #A32794; donkey anti‐goat Alexa Fluor Plus 647, #A32849; 1:1000) in DPBS with 0.1% BSA were incubated for 1 h at RT in the dark. Cells were washed 3× using DPBS with 0.05% Tween‐20 for 5 min each. Hoechst 33342 (20 µg/mL) was added during the second washing step for counterstaining. Cells were imaged using confocal microscopy.

### 4.10 Neuronal Differentiation

The neuronal differentiation protocol was adapted from previous work and used a doxycycline (DOX)‐inducible Neurogenin 2 (NGN2)‐expression cassette, featured in the hiPSC line [96]. Specifically, neuronal differentiation was initiated (0 DIV) from five‐day‐old hiPSC colonies by supplementing the mTeSR+ medium with 2 µg/mL DOX (Sigma Aldrich, #D9891). The next day (1 DIV), the medium was changed to N2B27+ medium (50% Neurobasal, ThermoFisher Scientific, #21103049; 50% DMEM/F12, ThermoFisher Scientific, #21331020; B27+ (50×, used as 100×), ThermoFisher Scientific, #A3582801; N2 (100×), ThermoFisher Scientific, #17502‐048; penicillin/streptomycin/L‐glutamine (100×), Sigma Aldrich, #G1146) supplemented with 2 µg/mL DOX. The next day (2 DIV), the predifferentiated colonies were dissociated using Accutase, and cells were seeded at 150 k cells/cm^2^ into Matrigel‐coated (2 h@37°C) 24‐well plates in N2B27+ medium supplemented with 2 µg/mL DOX. After 2–3 days (4–5 DIV in total), medium was replenished with fresh N2B27+ medium supplemented with 2 µg/mL DOX. Cells were fixed on days 5 and 7 for immunocytochemistry labeling (labeling procedure as described above).

### 4.11 Data Presentation, Analysis, and Statistics

Data are presented in means with data points; number of chip‐mediated transfection experiments (flat/blank, flat/mRNA, NTs/mRNA): each *n* = 3–8 (as indicated in the figure captions). Cell data extracted from images was obtained from 6 (viability, 3×2 = 6), 9 (pluripotency, 5 days, NANOG, OCT4, 3×3 = 9), 12 (pluripotency, passage 1–3, NANOG, OCT4, SOX2, 4×3 = 12), or 6 (neuronal differentiation, 3×2 = 3) images (∼580×580 µm^2^) per condition. For analysis, raw gray‐scale images were used. To increase readability in the manuscript, images were optimized in brightness and contrast, and presented in false color where applicable. Significance tests were conducted using one‐way ANOVA with post hoc Tukey's tests. Considered significances were ***: *p* < 0.001, **: *p* < 0.01, *: *p* < 0.05, and non‐significant (n.s.): *p* > 0.05. Data were analyzed using FlowJo (v10.10.0), CellProfiler (4.2.8) [98], and Origin Pro (2024b). Figures were arranged with Inkscape (1.4.3). Partly, graphics from biorender.com were used.

## Conflicts of Interest

The authors declare no conflicts of interest.

## Supporting information




**Supporting File**: adma72702‐sup‐0001‐SuppMat.pdf.

## Data Availability

The data that support the findings of this study are available in the supplementary material of this article.
