# Supplementary material for: Poking Pluripotency: Nanoinjection Into Human iPSCs
Source: Adv Mater. 2026 Mar 14;38(20):e21046. doi: 10.1002/adma.202521046 (PMC13054123; doi:10.1002/adma.202521046)
Supplement: Supplementary file 1 — Supporting File: adma72702‐sup‐0001‐SuppMat.pdf. [file ADMA-38-e21046-s001.pdf]

# Supporting information

## Poking Pluripotency: Nanoinjection into Human iPSCs

Jann Harberts*[,1,2], Vuong Thi Thanh Xuan Ho[1,2], Yuqi Yang[2,3], Yuan Jiang[4,5], Roey Elnathan*[,1,2,4,5,6], and Nicolas H. Voelcker*[,1,2,3]

[1]*Monash Institute of Pharmaceutical Sciences, Monash University, Parkville, VIC 3052, Australia*

[2]*Melbourne Centre for Nanofabrication, Victorian Node of the Australian National Fabrication Facility, Clayton, VIC 3168, Australia*

[3]*Materials Science and Engineering, Monash University, Clayton, VIC 3168, Australia*

[4]*School of Medicine, Faculty of Health, Deakin University, Waurn Ponds, VIC 3216, Australia*

[5]*The Institute for Mental and Physical Health and Clinical Translation, School of Medicine, Deakin University, Waurn Ponds, VIC 3216, Australia*

[6]*Institute For Frontier Materials, Deakin University, Waurn Ponds, VIC 3216, Australia*

E-mail: jann.harberts@monash.edu; roey.elnathan@deakin.edu.au; nicolas.voelcker@monash.edu

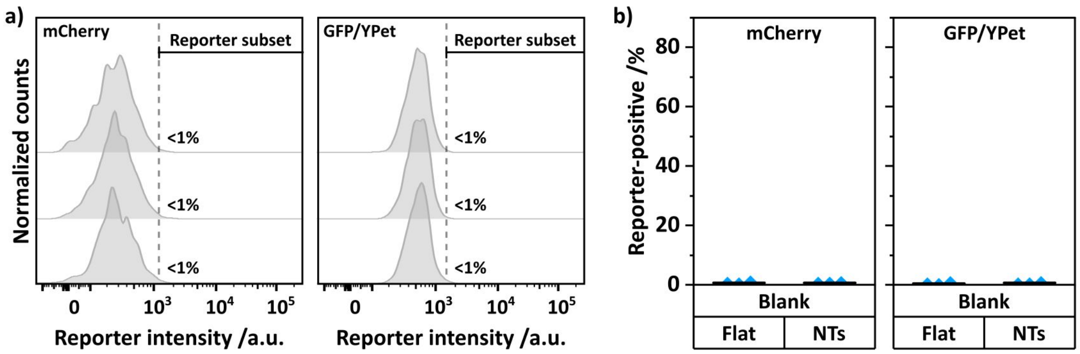


**Figure S1 — Nanoinjection without cargo (mCherry and GFP/YPet channel).** NTs without cargo showed no transfection. **a)** Intensity plots of reporter brightness. **b)** Quantification of reporter-positive cells. n = 3.

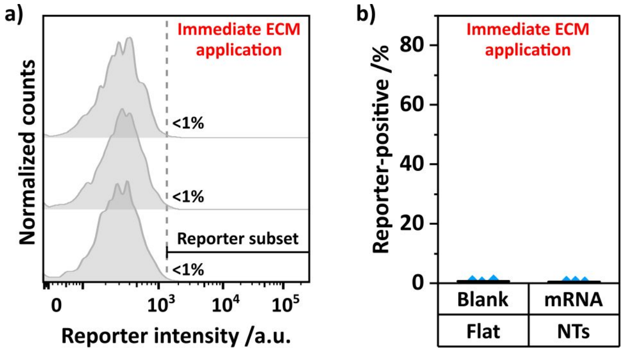


**Figure S2 — Impact of immediate ECM application.** When the ECM was applied immediately, no transfection was observed. **a)** Intensity plots of reporter brightness. **b)** Quantification of reporter-positive cells. n = 3.

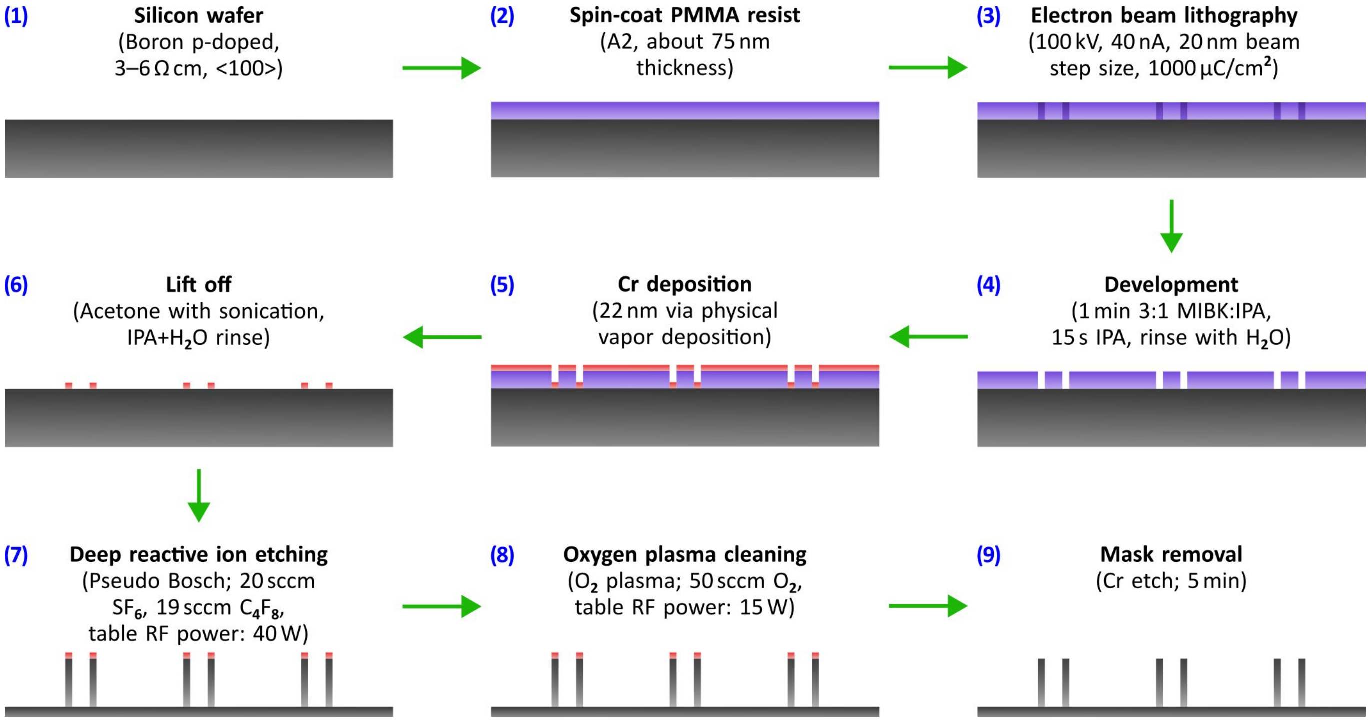


**Figure S3 — Fabrication steps used to prepare the NT arrays.** The NTs were fabricated from 4" silicon wafers (1). Wafers were spin-coated with ~75 nm PMMA (2). Ring patterns were defined using EBL (3). PMMA was developed to expose the rings (4). A 22 nm thick Cr layer to serve as a hard mark during etching was deposited using physical vapor deposition (5). Remaining PMMA was removed with an acetone lift off and only the Cr rings remained (6). NT structures were prepared using DRIE (7). The $C_4F_8$ passivation layer was removed with an oxygen plasma. Note, the oxygen plasma also reacts with Cr so that part of it may be removed in this step already (8). The Cr mask was removed to expose the sharp NT rims (9).

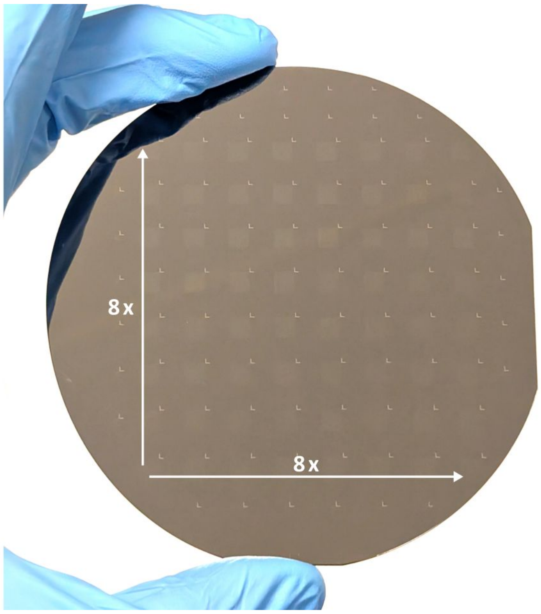


**Figure S4 — Example of the wafer scale patterning.** Each 4" wafer was exposed with 8×8 pattern of 4×4 mm$^2$. Spare areas outside the 8×8 grid were used for test pattern.

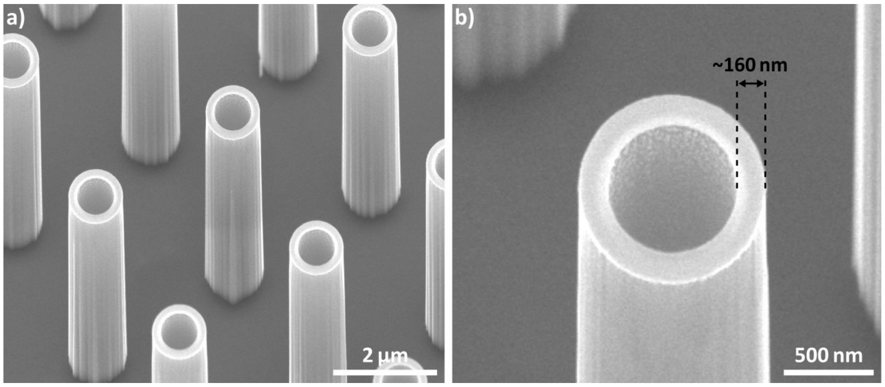


**Figure S5 — Exemplary SEM images of NTs still containing the Cr mask. a)** Overview SEM image of NTs still with the Cr mask at the NT tip, i.e., before Cr removal. **b)** Close-up image of the NT tip. The Cr ring had a width of about 160 nm.

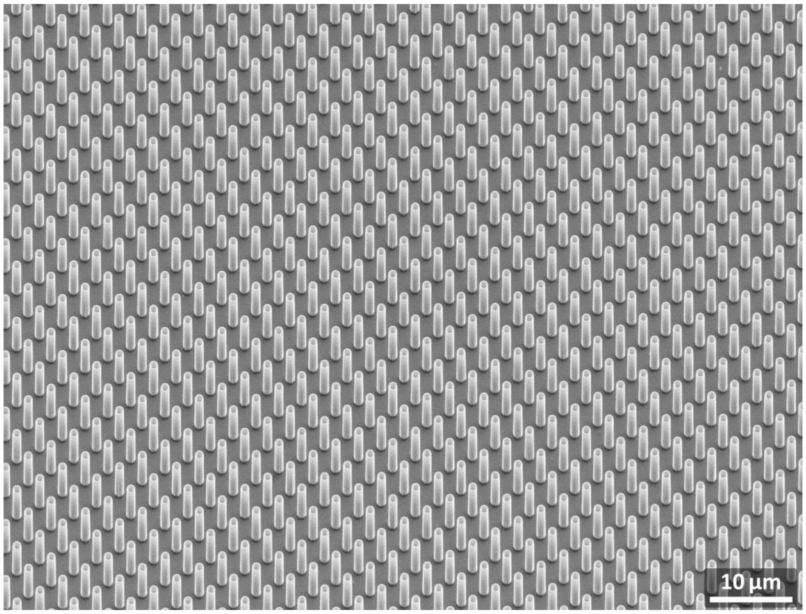


**Figure S6 — Overview SEM image of NT arrays.** The NTs were fabricated with high uniformity across the patterned area.

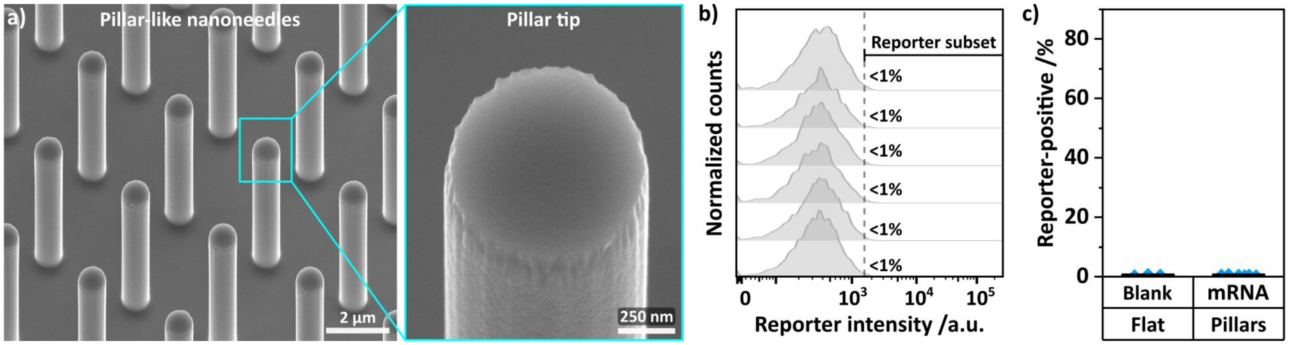


**Figure S7 — Nanoinjection with pillar-like nanoneedles, i.e., without cavity and sharp rim.** Without cavity and sharp rim, no transfection was observed. **a)** SEM image of a pillar-like nanoneedles, including zoom at the pillar tip. **b)** Intensity plots of hiPSCs nanoinjected with pillar-like nanoneedles. **c)** Proportions of reporter-positive hiPSCs using pillar-like nanoneedles. n = 6.

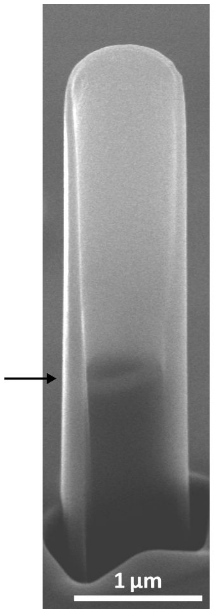


**Figure S8 — Exemplary cross-sectional SEM image of a NT prepared with FIB milling.** The NT reservoir accounted for about 75% of the NT height (indicated by the arrow). The walls featured a tapered thickness profile, narrowing toward the tip of the NT.

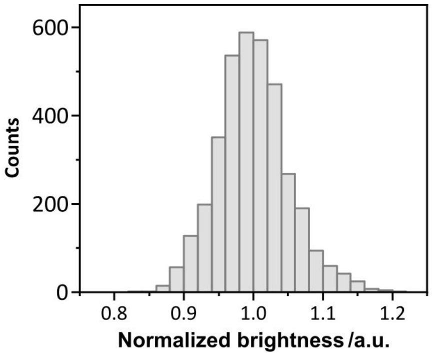


**Figure S9 — Analysis of cargo loading uniformity.** Normalized brightness distribution of NTs loaded with Cy5-tagged mRNA. n = 3604 NTs, SD = 0.052, min = 0.83, max = 1.21.

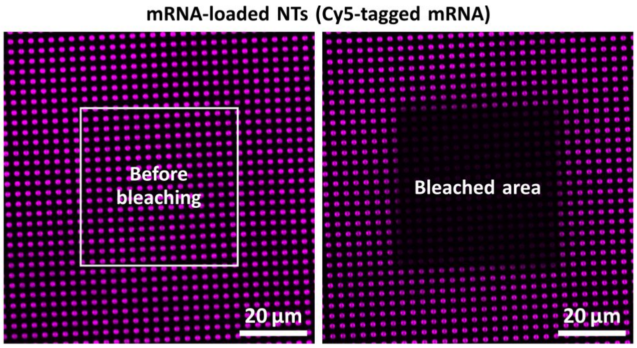


**Figure S10 — Confirming the signal origin: bleaching test of Cy5-tagged mRNA loaded NTs.** Bleaching test to confirm that the recorded signal originated from the Cy5 dye but not from substrate reflections. Before bleaching (homogeneous dot pattern) and after bleaching (Cy5 signal in the exposed area vanishes).

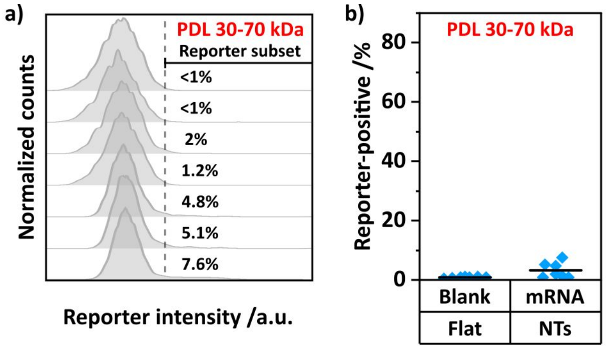


**Figure S11 — Nanoinjection using PDL with a high mol. wt. of 30–70 kDa (PDL70) for the surface functionalization: transfection was minimal.** **a)** Intensity histograms using PDL70. **b)** Proportion of reporter-positive hiPSCs using PDL70. n = 7.

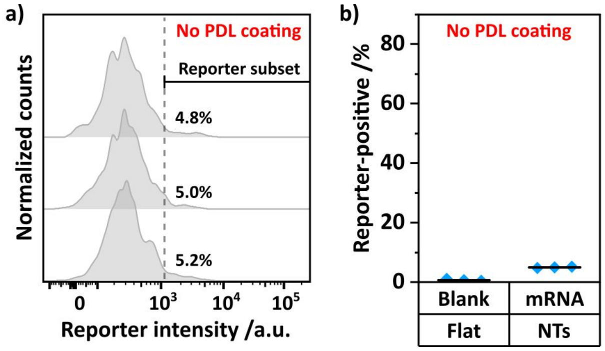


**Figure S12 — Nanoinjection without PDL functionalization: transfection was minimal.** **a)** Intensity histograms without PDL. **b)** Proportion of reporter-positive hiPSCs without PDL. n = 3.

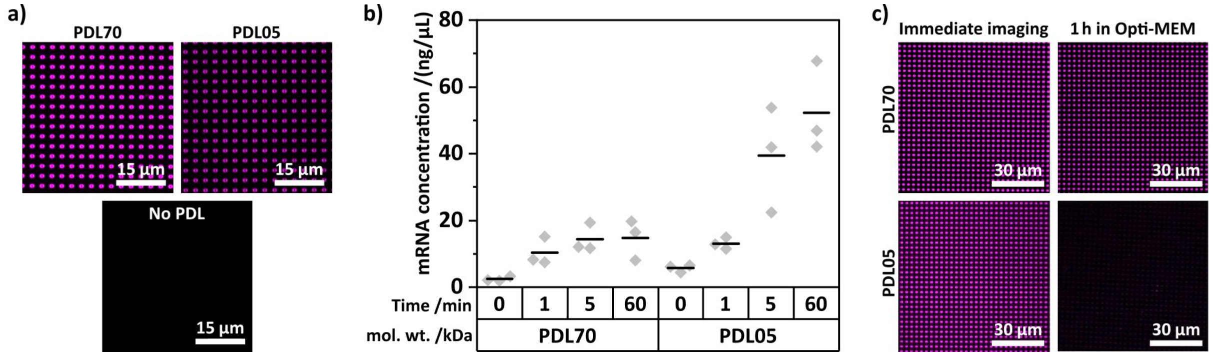


**Figure S13 — mRNA loading (Cy5-tagged) and release using different mol.wt. PDLs (PDL05: 1–5 kDa, PDL70: 30–70 kDa) and no PDL.** **a)** Confocal images of loading with PDL70, PDL05, and no PDL. At same laser power, PDL70 showed stronger Cy5 signal than PDL05, indicating larger amounts of mRNA loaded. No PDL showed no Cy5 signal. **b)** mRNA release from chips coated with PDL70 and PDL05 after 0, 1, 5, and 60 min. For PDL70, the released cargo amount was minimal despite high initial loading. For PDL05, the amount of released mRNA increased over time. **c)** Confocal images of Cy5 signal signal after 1 h storage in Opti-MEM compared to initial brighness (laser power adjusted to maximal brightness at the beginning for both PDL types.) For PDL70, Cy5 signal remained after 1 h. For PDL05, the Cy5 signal vanished.

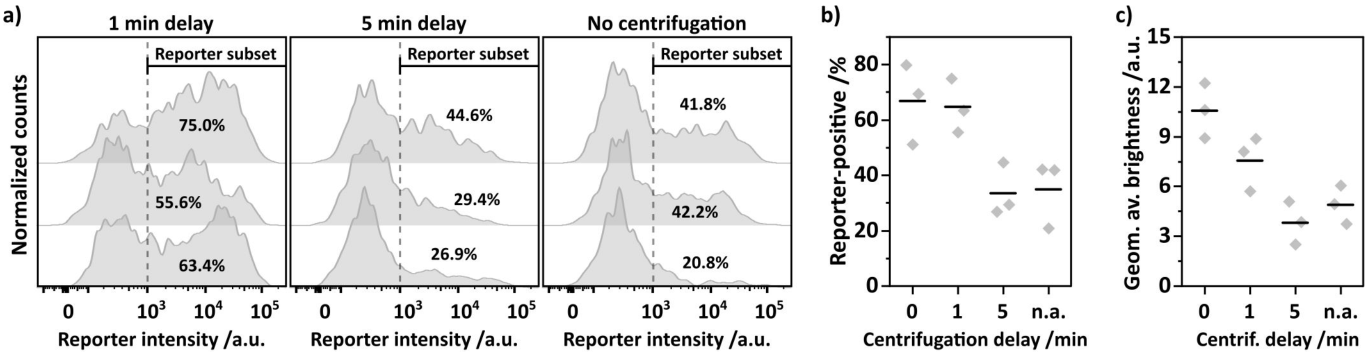


**Figure S14 — Impact of delaying (1 and 5 min) or skipping (n.a.) the centrifugation step. a)** Intensity histogram of hiPSCs nanoinjected with a delayed centrifugation step and without centrifugation, mCherry (Messenger Bio). **b)** Proportion of reporter-positive hiPSCs nanoinjected with a delayed/skipped centrifugation step. **c)** Brightness of reporter-positive hiPSCs. n = 3.

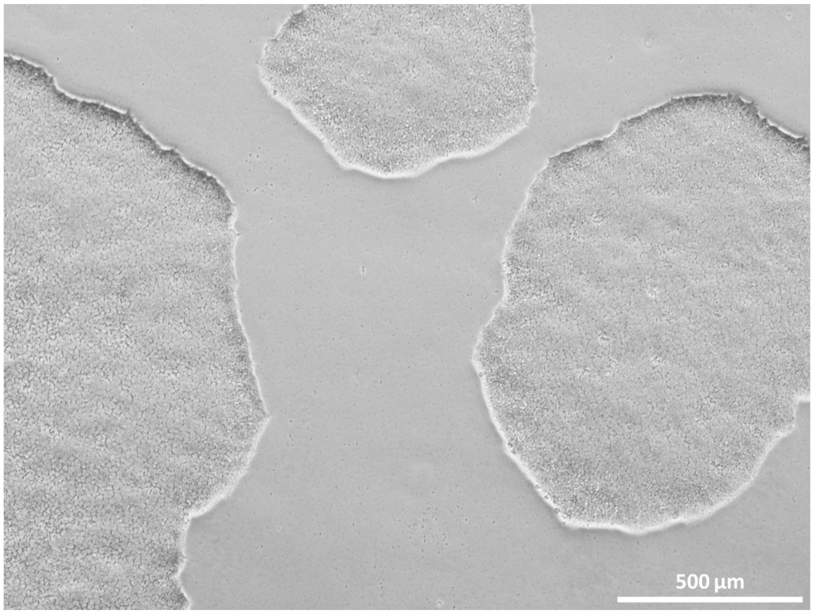


**Figure S15 — Representative brightfield image of the hiPSC colonies.** The hiPSCs grew in dense colonies featuring well-defined colony edges and no spontaneous differentiation.

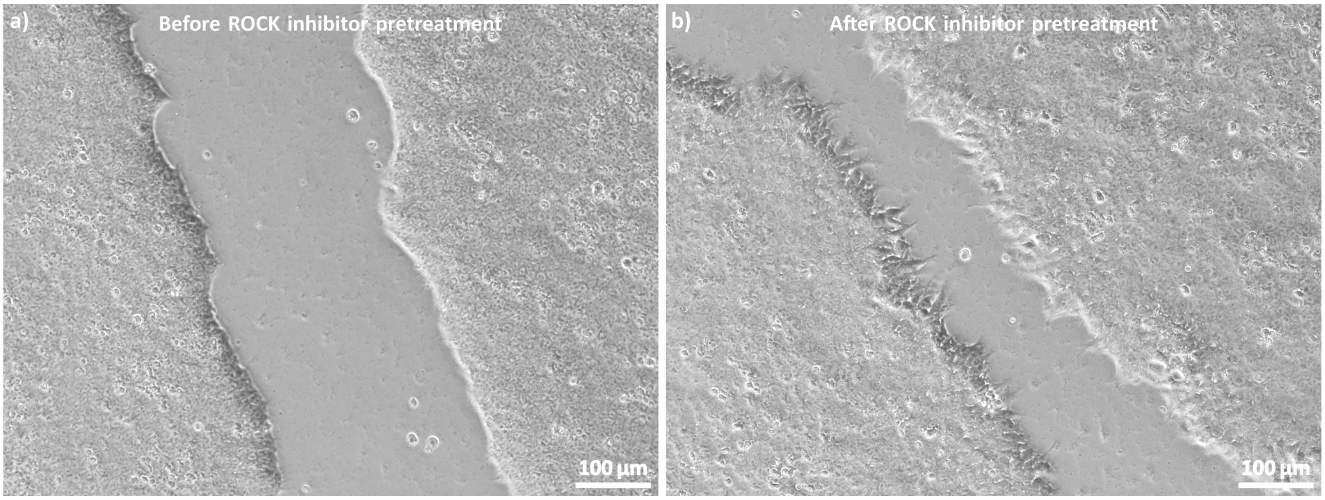


**Figure S16 — Representative brightfield images of hiPSC colonies without and with ROCK inhibitor pretreatment. a)** Untreated hiPSC colonies exhibited smooth, compact edges. **b)** ROCK inhibitor–treated hiPSC colonies showed roughened edges.

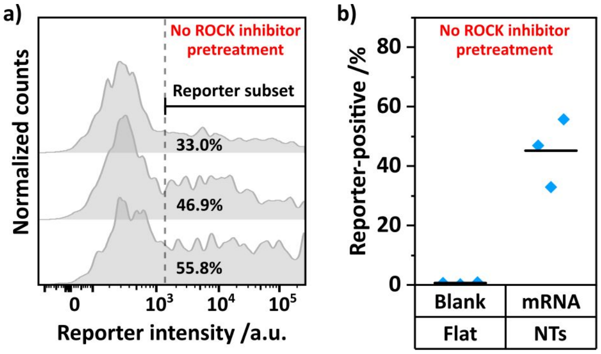


**Figure S17 — Nanoinjection without ROCK inhibitor pretreatment.** Without ROCK inhibitor pretreatment, transfection was feasible but less reliable. n = 3.

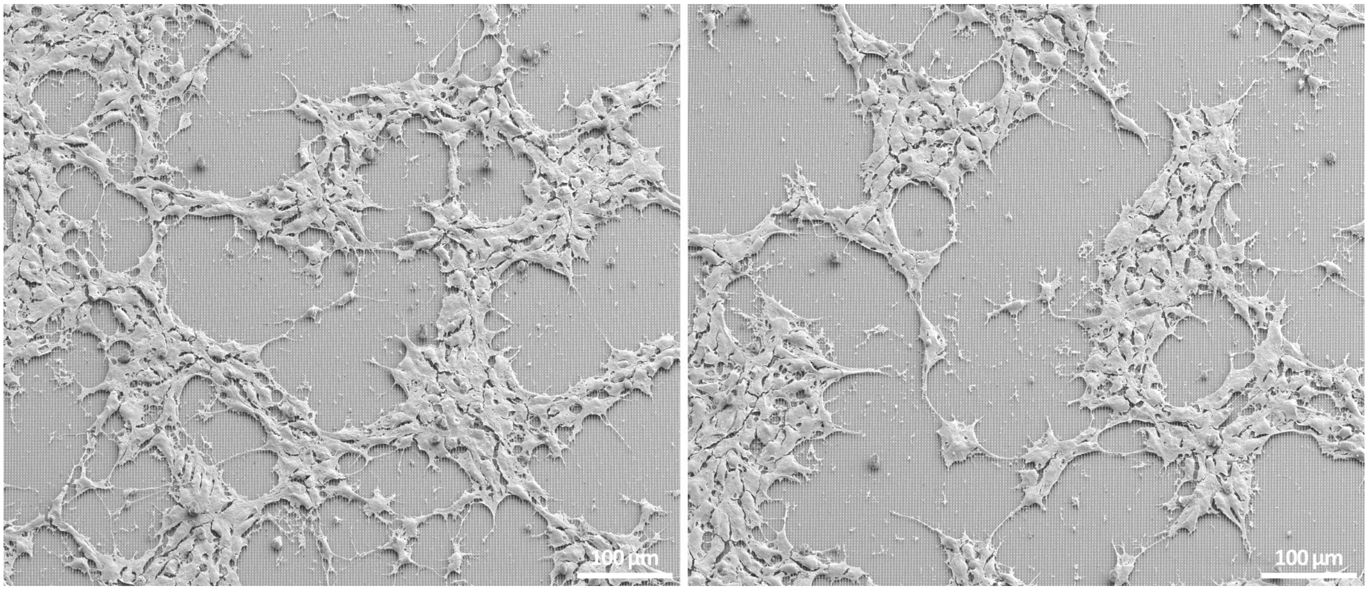


**Figure S18 — Exemplary overview SEM images of cells interfaced with NT arrays.** The hiPSCs spread across the NT array.

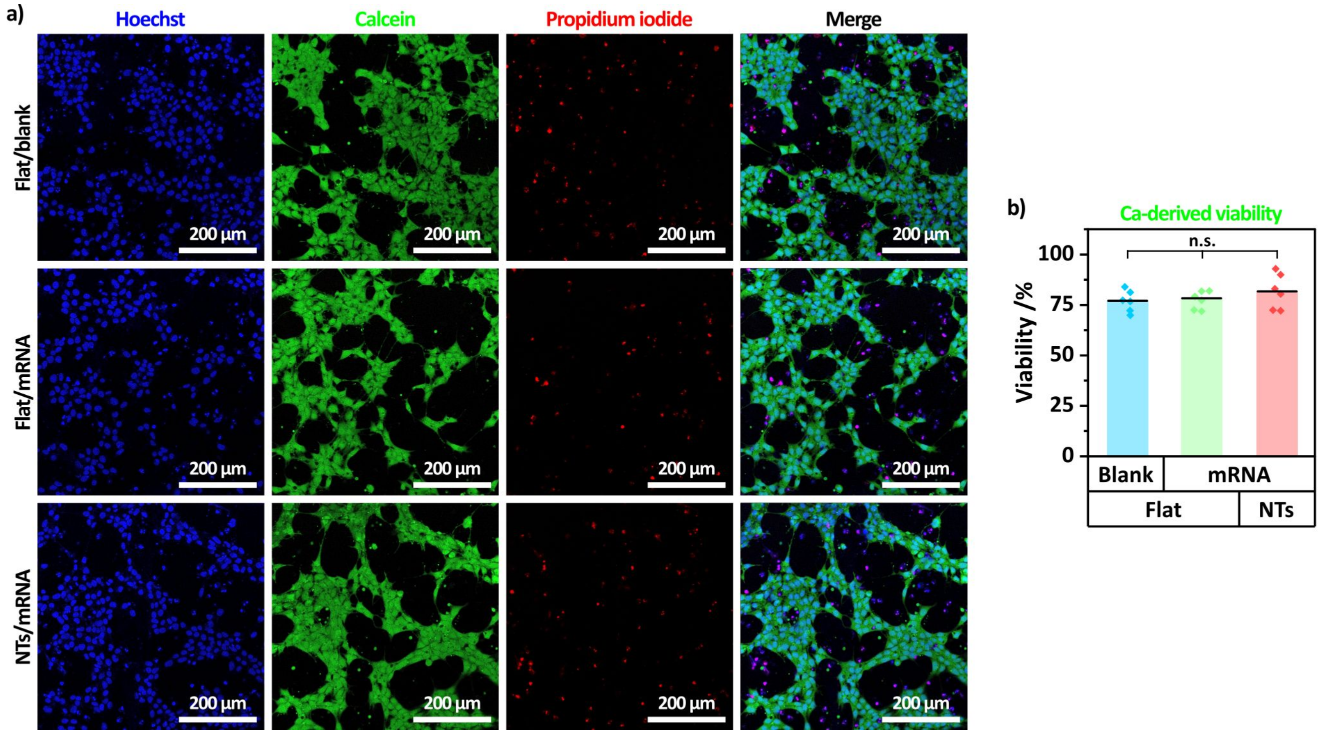


**Figure S19 — Viability of hiPSCs interfaced with the NTs. a)** Exemplary confocal microscopy images of hiPSCs cultured overnight (i.e., just before harvest) on flat/blank, flat/mRNA, and NTs/mRNA stained with Hoechst (nuclei), Calcein (Ca, viable cells), and propidium iodide (PI, dead cells). **b)** Viability of hiPSCs cultured overnight on the chips derived from Ca (Ca-positive nuclei). ANOVA with post hoc Tukey's test, n = 3.

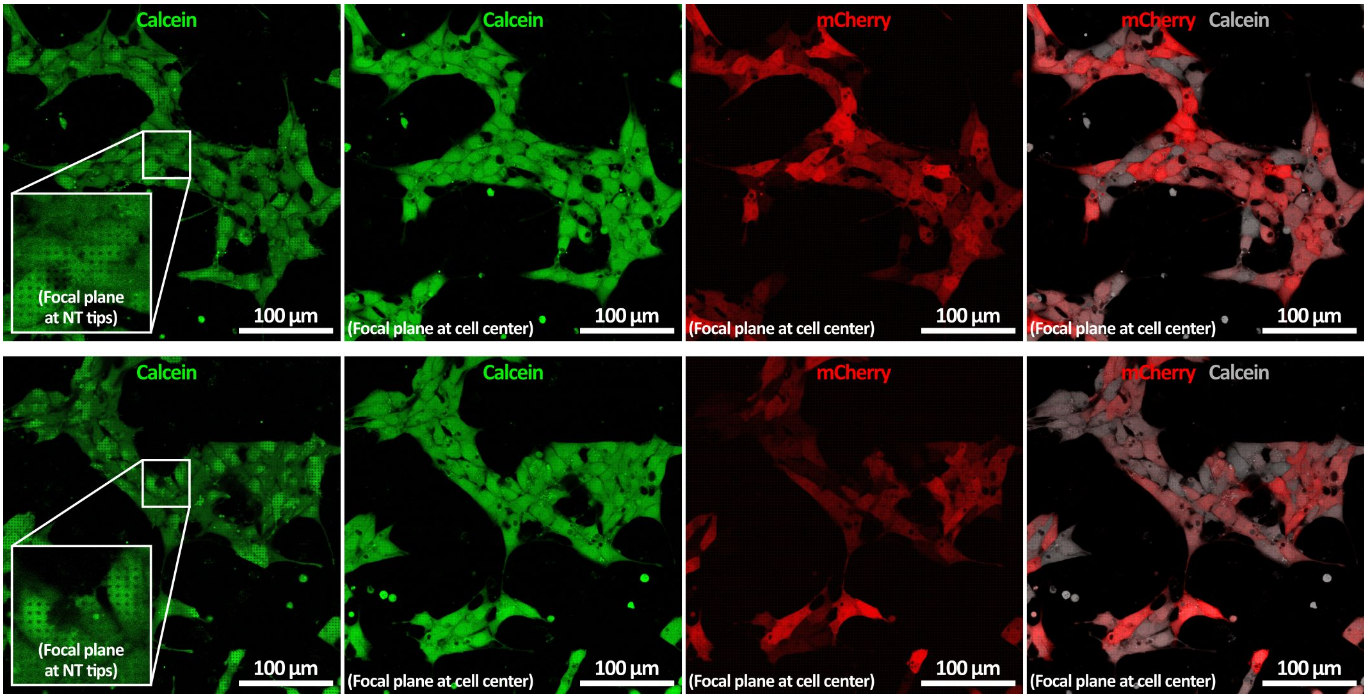


**Figure S20 — Additional exemplary images of mCherry-expressing hiPSCs interfaced with NTs.** The positions of the NTs were visible (dark spot pattern) in the Calcein channel (see magnified inset, focal plane at NT tips; other panels: focal plane at cell center, further away from the NTs).

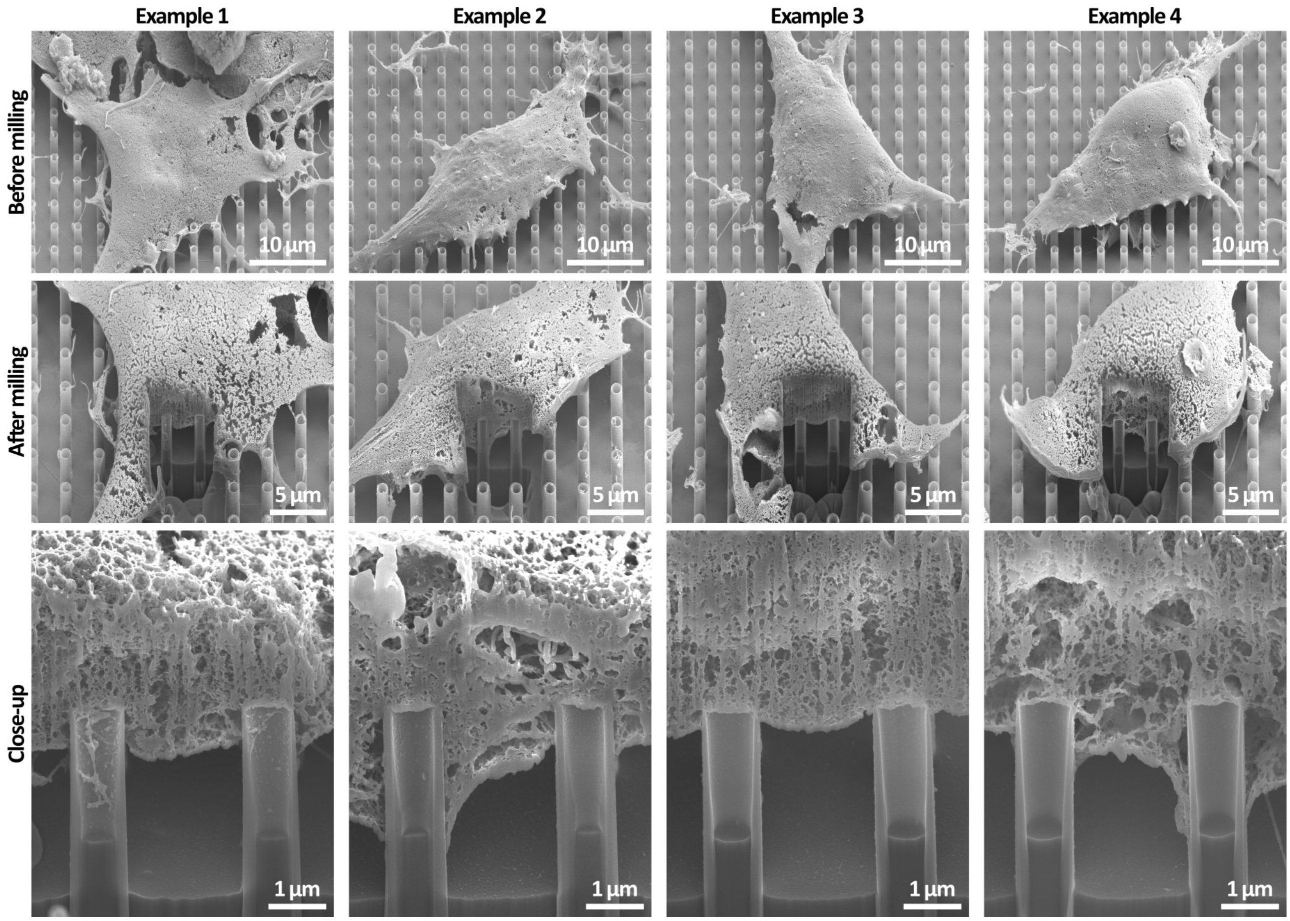


**Figure S21 — Exemplary cross-sectional SEM images prepared by FIB milling.** The cells rested on the NT tips, encapsulating only the upper part of the NTs without contact with the substrate bottom.

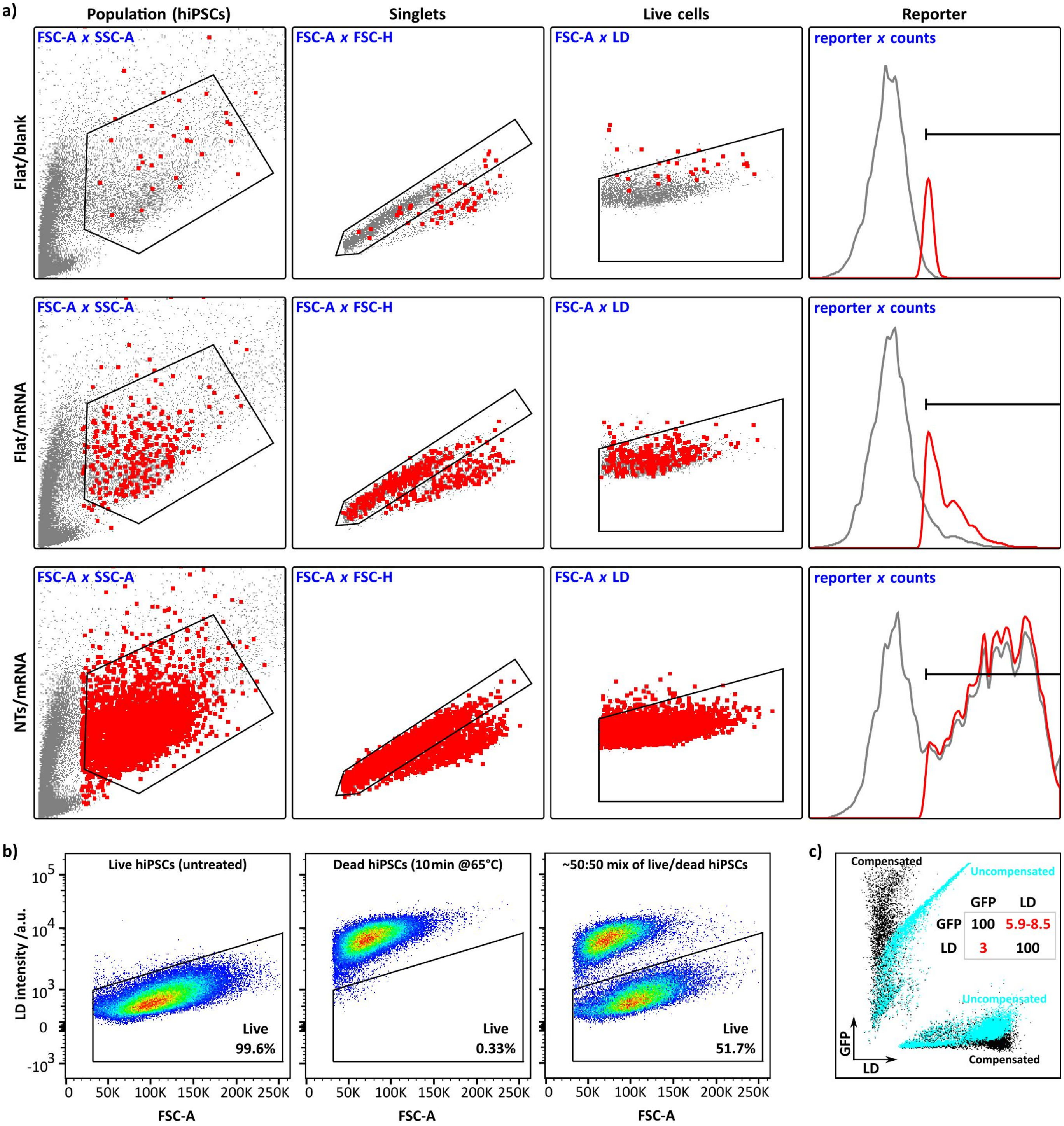


**Figure S22 — Gating strategy for flow cytometry analysis, including back-gating of reporter-positive cells (large red dots), LD control, and LD/GFP compensation. a)** Gating stream: population by size and granularity (FSC-A×SSC-A), singlets by size and shape correlation (FSC-A×FSC-H), live cells by excluding LD-positive cells (FSC-A×LD), and reporter intesity histograms (reporter×counts). Reporter-positive cells showed a homogeneous distribution in the FSC-A×SCC-A plot, indicating non-selective transfection of the hiPSCs. **b)** LD staining control: live hiPSCs (untreated), dead hiPSCs (10 min at 65°C), ∼50:50 mixture of live and dead hiPSCs. **c)** Compensation of GFP and LD. Exemplary scatter plot of LD- and GFP-positive hiPSCs without compensation (turquise) and with compensation (black), respectively, including the applied compensation matrix (notably, cross talk from YPet into LD was minimal: ≤0.5%).

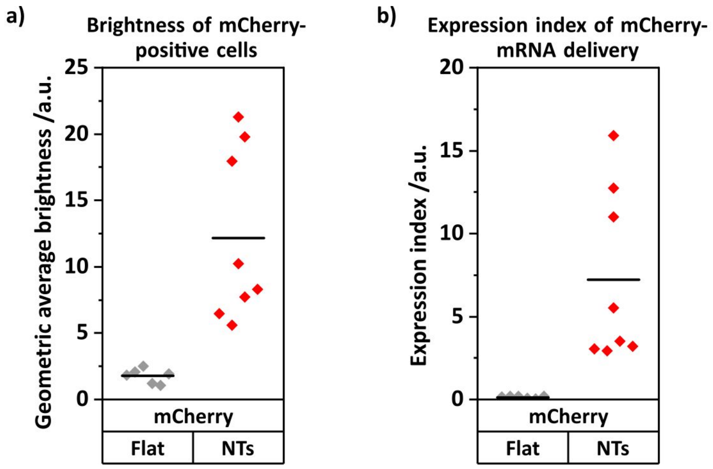


**Figure S23 — Additional flow cytometry analysis.** **a)** Brightness of mCherry-positive cells (minus gating brightness). **b)** Expression index of mCherry-mRNA delivery, (brightness − gating brightness)×(yield − gating yield). n = 8.

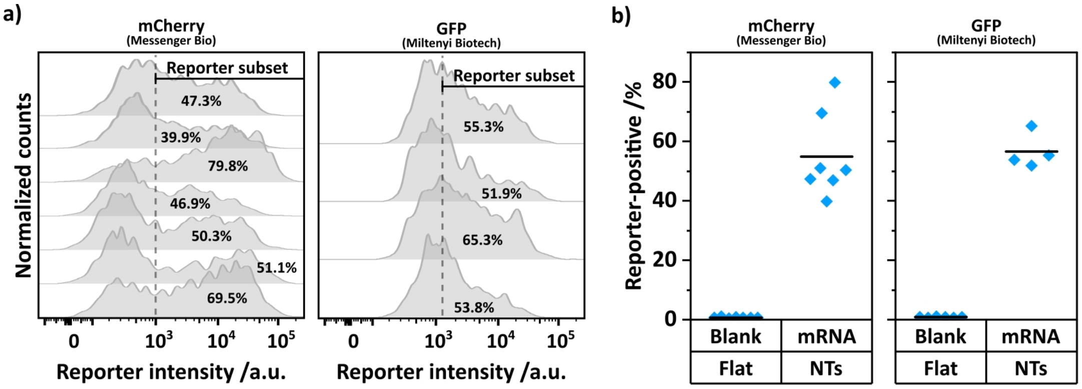


**Figure S24 — Nanoinjection using different mCherry and GFP mRNA (Messenger Bio, Miltenyi Biotech).** **a)** Intesity plots of mCherry and GFP. **b)** Proportion of mCherry- and GFP-positive hiPSCs. n = 4–7.

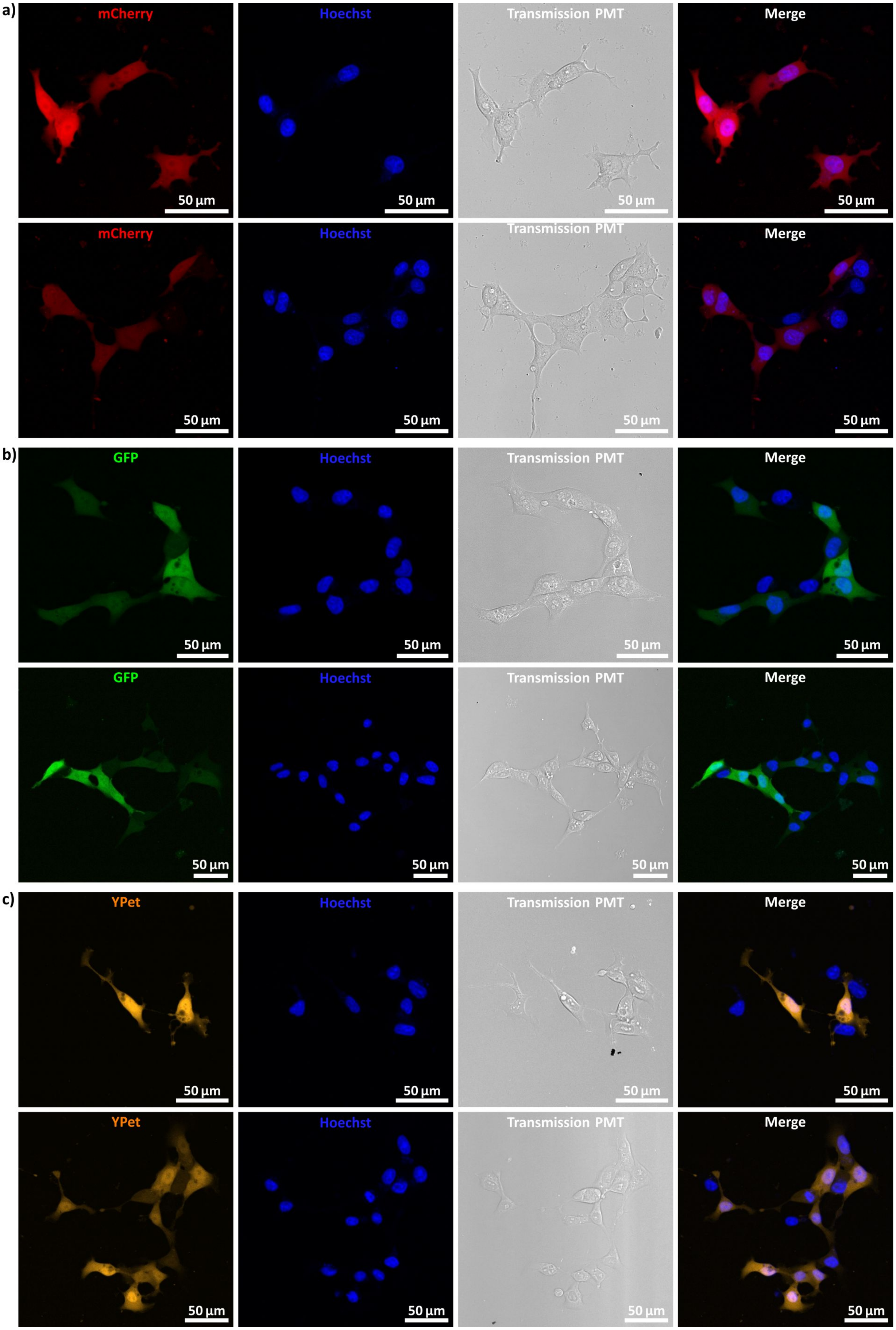


**Figure S25 — Additional exemplary images of reseeded nanoinjected hiPSCs (mCherry, GFP, and YPet).** The hiPSCs showed homogeneous reporter signal within the cytosol with varying brightnesses across individual cells. **a)** mCherry. **b)** GFP. **c)** YPet.

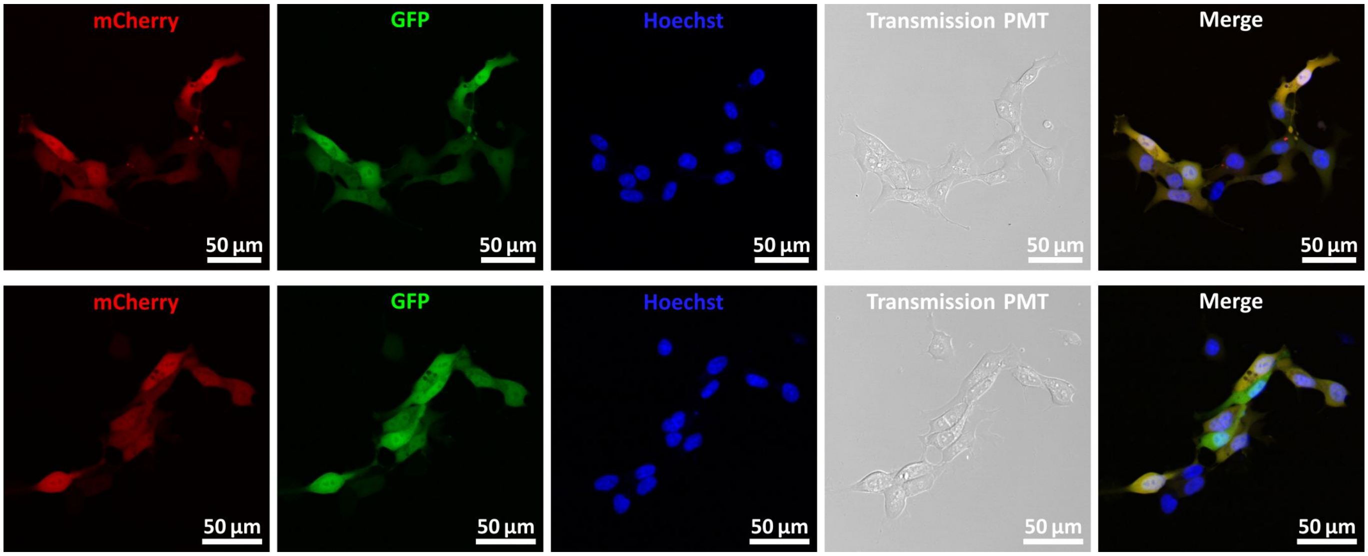


**Figure S26 — Additional exemplary images of reseeded co-nanoinjected hiPSCs (mCherry+GFP).** The hiPSCs showed homogeneous mCherry and GFP signal within the cytosol with varying brightnesses across individual cells. The merge shows the cells expressing both mRNA types.

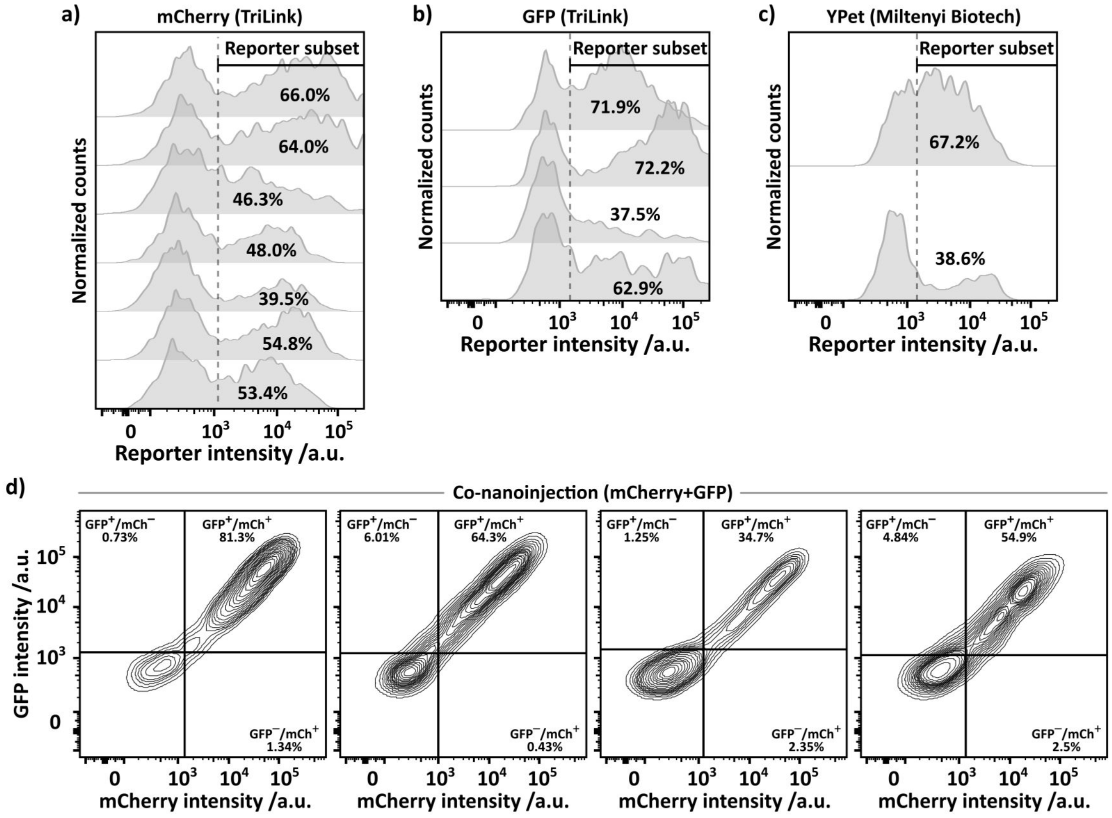


**Figure S27 — Compilation of all reporter histograms used for analysis but not shown elsewhere.** **a)** mCherry plots (used in Figure 4b). **b)** GFP plots (used in Figure 4d). **c)** YPet plots (used in Figure 4d). **d)** Co-nanoinjection plots (used in Figure 4g).

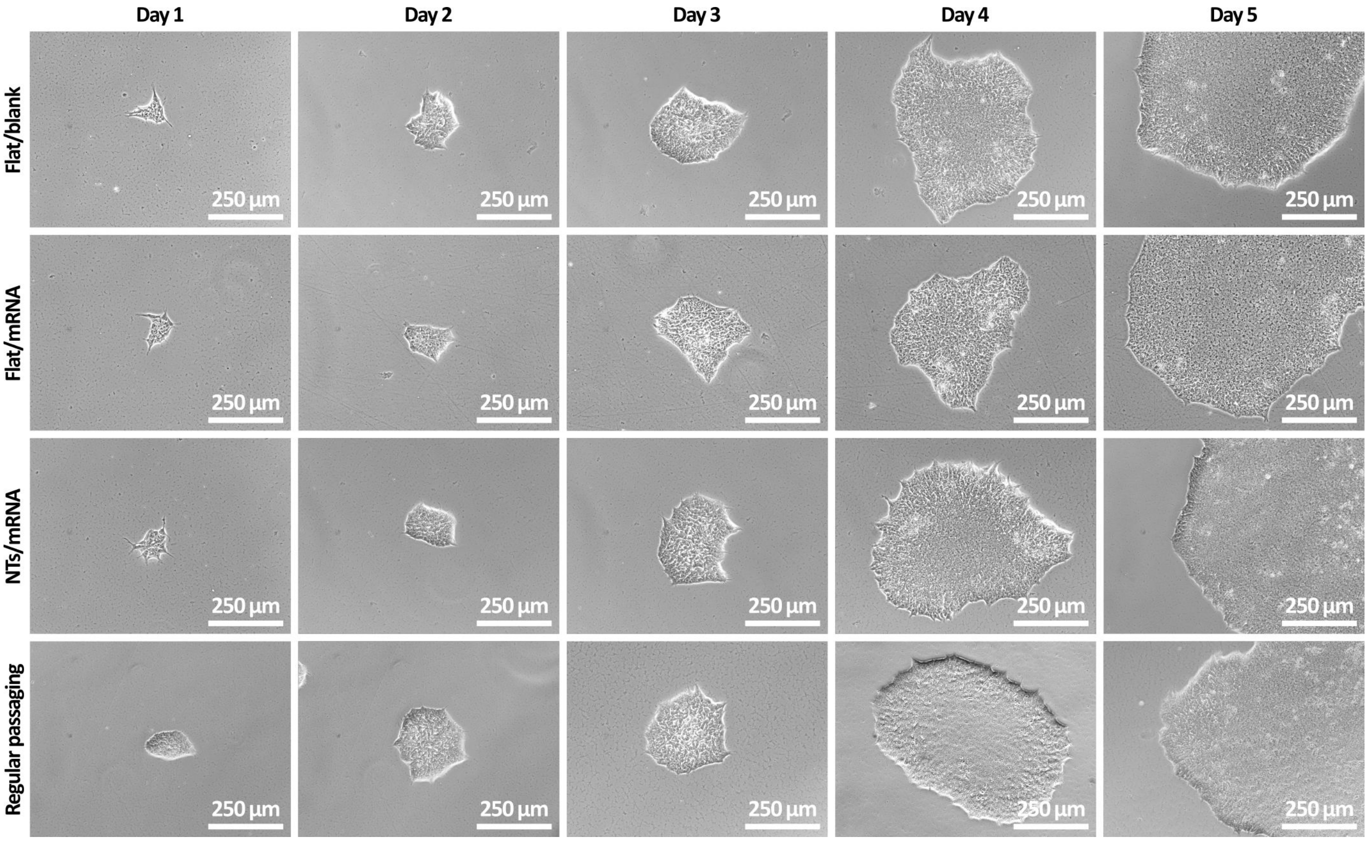


**Figure S28 — Exemplary brightfield images of the colony formation of hiPSCs harvested from NTs and controls.** Colony formation was imaged daily (1–5 days) for hiPSCs harvested from flat/blank, flat/mRNA, and NTs/mRNA as well as hiPSCs obtained through regular passaging in small cell clusters (using ReLeSR, no ROCK inhibitor at day 1). The colonies showed compact growth with defined edges. n = 3.

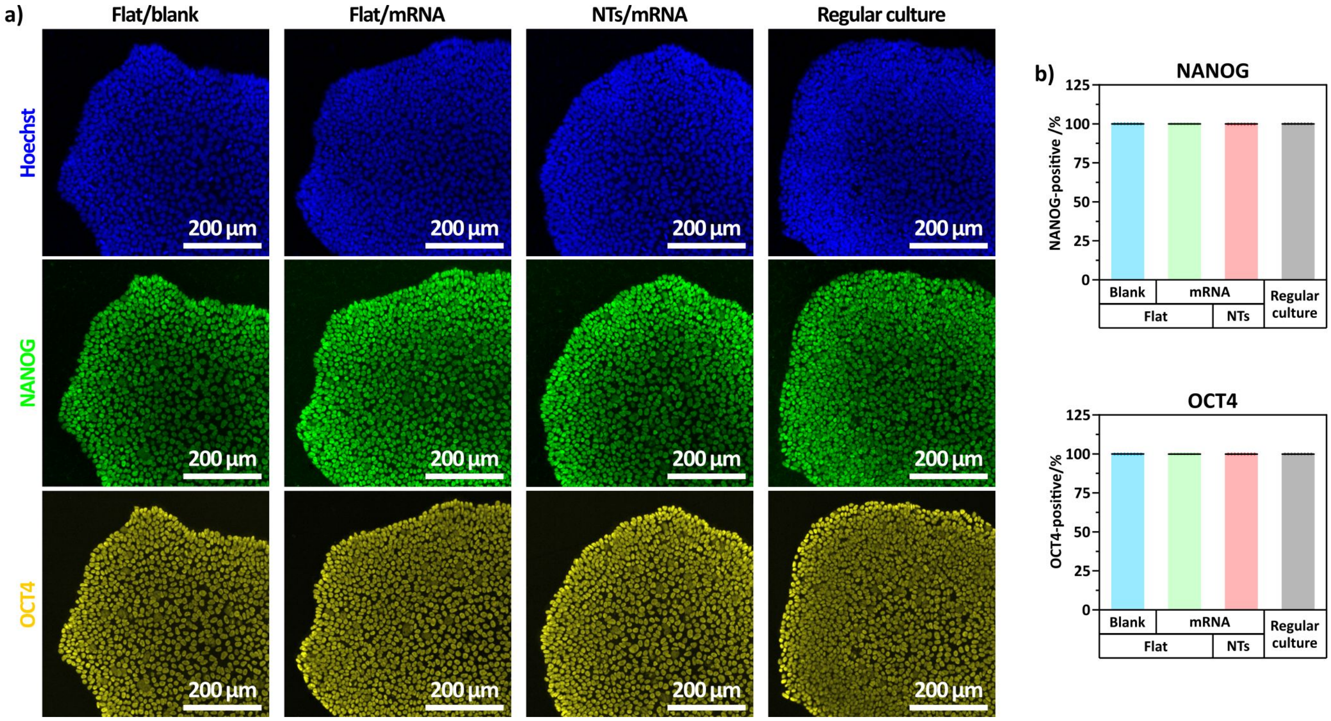


**Figure S29 — Immunocytochemistry staining of pluripotency markers (NANOG, OCT4) of harvested and reseeded hiPSCs (5 days of culture). a)** Exemplary NANOG and OCT4 images of hiPSCs harvested from flat/blank, flat/mRNA, and NTs/mRNA, including regularly passaged hiPSCs. Nuclei were counterstained with Hoechst 33342. **b)** Quantification of NANOG- and OCT4-positive cells. n = 3.

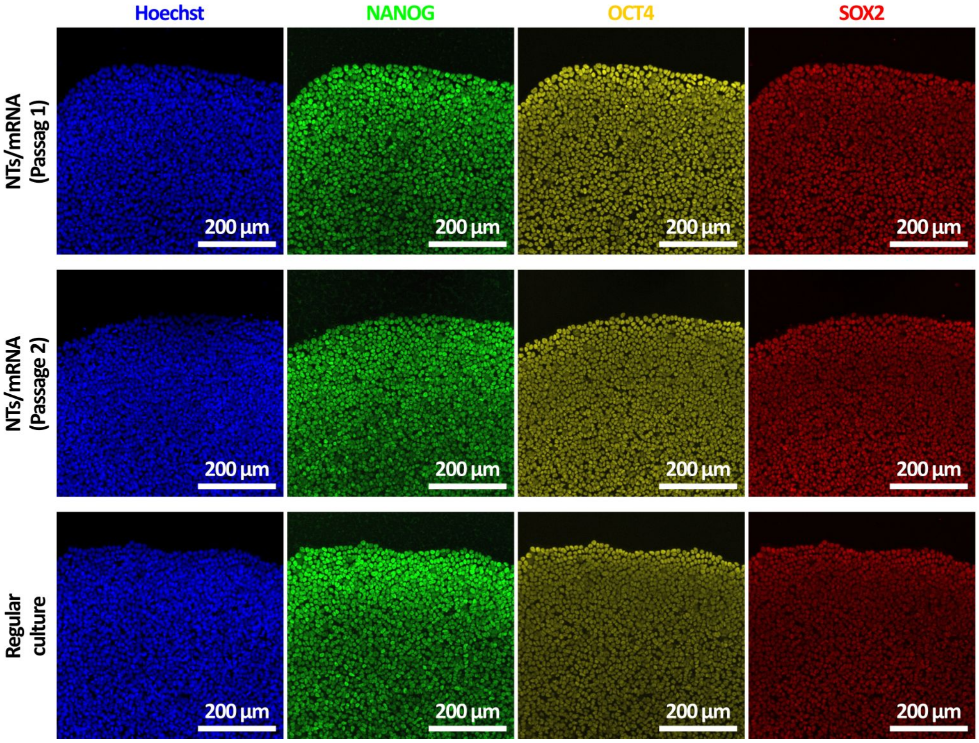


**Figure S30 — Immunocytochemistry staining of pluripotency markers (NANOG, OCT4, SOX2) of harvested and reseeded hiPSCs monitored for three passages (~3 weeks).** Exemplary NANOG, OCT4, and SOX2 images of hiPSCs harvested from NTs/mRNA (passage 1 and 2, passage 3 shown in the main manuscript), including regularly cultured hiPSCs. Nuclei were counterstained with Hoechst 33342. n = 4.

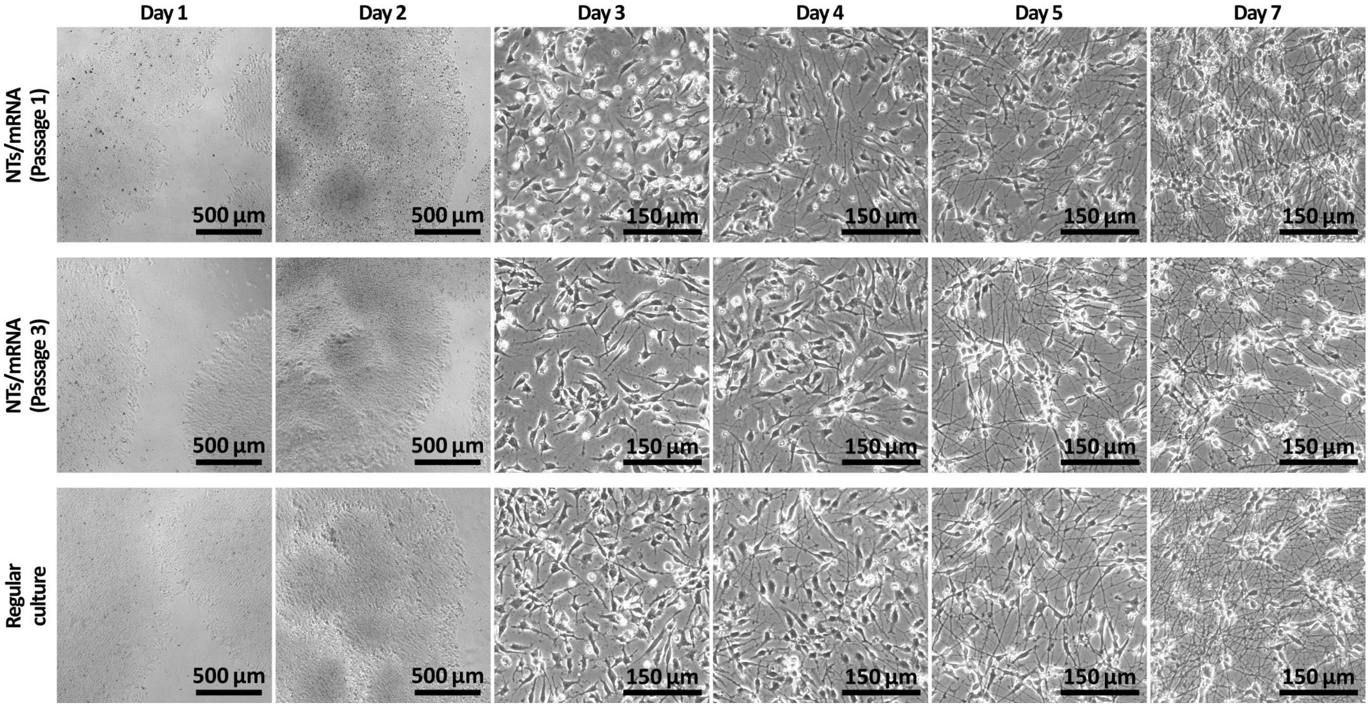


**Figure S31 — Exemplary brightfield images of the morphological changes during neuronal differentiation using hiPSCs recovered after nanoinjection.** Neuronal differentiation was conducted from hiPSCs 1 and 3 passages after nanoinjection and compared to differentiation from regularly cultured hiPSCs. Cells were imaged at day 1, 2, 3, 4, 5, and 7. A colony-initiated differetiation protocol was used, where colonies formed the expected substructes at day 2 (before replating) and cells formed neurons within 7 days. n = 3.

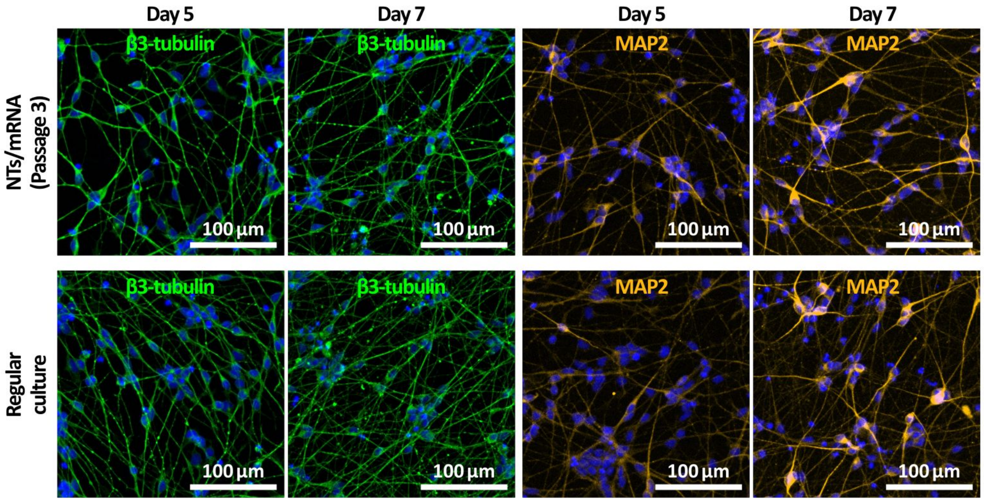


**Figure S32 — Exemplary immunocytochemistry stainings of neurons labeled for β3-tubulin and MAP2 (day 5 and 7).** Displayed are neurons generated from nanoinjected hiPSCs three passages after transfection and controls using regularly passaged hiPSCs (neuronral differentiation of passage 1 nanoinjected hiPSCs in the main manuscript.) Neurons are positive for β3-tubulin and MAP2. n = 3.

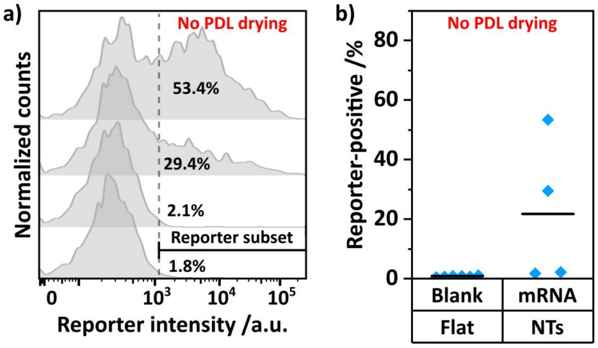


**Figure S33 — Impact of not-drying the PDL during functionalization.** When the PDL was not dried, transfection was reduced and inconsistent. **a)** Intensity plots of reporter brightness. **b)** Quantifcation of reporter-positive cells. n = 4.
